# Additive Expression of Consolidated Memory through *Drosophila* Mushroom Body Subsets

**DOI:** 10.1371/journal.pgen.1006061

**Published:** 2016-05-19

**Authors:** Chu-Huai Yang, Meng-Fu Maxwell Shih, Ching-Ching Chang, Meng-Hsuan Chiang, Hsiang-Wen Shih, Ya-Lun Tsai, Ann-Shyn Chiang, Tsai-Feng Fu, Chia-Lin Wu

**Affiliations:** 1 Graduate Institute of Biomedical Sciences, College of Medicine, Chang Gung University, Taoyuan, Taiwan; 2 Institute of Biotechnology, National Tsing Hua University, Hsinchu, Taiwan; 3 Department of Biochemistry, College of Medicine, Chang Gung University, Taoyuan, Taiwan; 4 Department of Applied Chemistry, National Chi-Nan University, Nantou, Taiwan; 5 Department of Neurology, Linkou Chang Gung Memorial Hospital, Taoyuan, Taiwan; Katholieke Universiteit Leuven, BELGIUM

## Abstract

Associative olfactory memory in *Drosophila* has two components called labile anesthesia-sensitive memory and consolidated anesthesia-resistant memory (ARM). Mushroom body (MB) is a brain region critical for the olfactory memory and comprised of 2000 neurons that can be classified into αβ, α′β′, and γ neurons. Previously we demonstrated that two parallel pathways mediated ARM consolidation: the serotonergic dorsal paired medial (DPM)–αβ neurons and the octopaminergic anterior paired lateral (APL)–α′β′ neurons. This finding prompted us to ask how this composite ARM is retrieved. Here, we showed that blocking the output of αβ neurons and that of α′β′ neurons each impaired ARM retrieval, and blocking both simultaneously had an additive effect. Knockdown of *radish* and *octβ2R* in αβ and α′β′ neurons, respectively, impaired ARM. A combinatorial assay of *radish* mutant background *rsh*^*1*^ and neurotransmission blockade confirmed that ARM retrieved from α′β′ neuron output is independent of *radish*. We identified MBON-β2β′2a and MBON-β′2mp as the MB output neurons downstream of αβ and α′β′ neurons, respectively, whose glutamatergic transmissions also additively contribute to ARM retrieval. Finally, we showed that α′β′ neurons could be functionally subdivided into α′β′m neurons required for ARM retrieval, and α′β′ap neurons required for ARM consolidation. Our work demonstrated that two parallel neural pathways mediating ARM consolidation in *Drosophila* MB additively contribute to ARM expression during retrieval.

## Author Summary

One of tantalizing questions in neuroscience is how the brain processes memory. Studies in animal models such as fruit fly have brought innovations addressing the general principles underlying memory processing such as acquisition, consolidation, and retrieval. Here, we revealed an additive expression of aversive consolidated memory through fly mushroom body (MB) subsets. By thermogenetic blockade of neurotransmission, we identified the necessity of MB αβ and α′β′ neurons, and their respective downstream neurons, for consolidated memory retrieval. We also showed that MB αβ and α′β′ neurons harbor distinct signaling pathways for memory consolidation by genetic manipulation. Notably, the combinatorial assays of neurotransmission blockade and genetic manipulations confirmed the independency between the two sets of double-layered parallel circuits. Our work, together with previous finding of two respective modulatory neurons upstream of αβ and α′β′ neurons, favor the notion that memory is consolidated in different brain regions/circuits in parallel and later additively retrieved for behavioral outcome.

## Introduction

Memory expression requires sequential processing such as acquisition, consolidation, and retrieval. The fruit fly (*Drosophila melanogaster*) is of great interest to neuroscientists studying memory because of its short lifespan, relatively simple brain, and powerful genetic tools. In fly aversive olfactory conditioning, the association between the electric shock and odor identity is first registered in odor-responsive MB γ neurons by dopamine signaling [1]. After acquisition, memory can be dichotomized to anesthesia-sensitive memory (ASM) and anesthesia-resistant memory (ARM) depending on the susceptibility to retrograde amnesia, and 3-h memory comprises equal extent of ASM and ARM [2–4]. ARM has been seen as a stable consolidated memory less costly than long-term memory and can be assessed by cold-induced anesthetization [4–6]. Many lines of evidence from gene mutation, RNA interference (RNAi)-mediated knockdown, and manipulation of neuronal activity support a model in which ASM and ARM constitute two independent types of memory that record the same episode [7, 8]. Here, we focus on deciphering the ARM processing.

The *Drosophila* MB, a paired neuropil structure that consists of ~2000 neurons, can be divided into subsets of neurons that comprise the αβ, α′β′, and γ lobes [9, 10]. The cell bodies of the MB neurons reside near the dorsal posterior surface of the protocerebrum. The dendrites of MB neurons form the calyx, where they receive olfactory input from the antennal lobe and transform it into a sparse neural code that benefits the establishment of associations between conditioned and unconditioned stimuli [11–14]. The axons of MB neurons project anteriorly to form a stalk-like pedunculus before branching into the vertical and horizontal lobes. The bifurcated axons of the αβ neurons form the α and β lobes of the vertical and horizontal lobes, respectively, and those of the α′β′ neurons form their corresponding α′ and β′ lobes. In contrast, the axons of the γ neurons constitute the horizontal γ lobe. According to the laminar zones within each MB lobes, α/β lobes have been further divided into core, surface, and posterior strata, while α′/β′ lobes have been divided into anterior, middle, and posterior strata [15]. Recently, Aso et al. used split-GAL4 screen and single-cell imaging to divide the MB neurons into seven cell types: αβp, αβs, αβc, α′β′ap, α′β′m, γmain, and γd [16, 17].

ARM requires *bruchpilot* expression in αβ neurons and acute *radish* expression right before training [2, 18, 19]. Two pairs of MB modulatory neurons, dorsal paired medial (DPM) and anterior paired lateral (APL) neurons, broadly innervating the MB also contribute to ARM formation by the serotonergic neurotransmission toward αβ neurons and the octopaminergic toward α′β′ neurons, respectively [15, 20–22], suggesting the presence of two parallel circuits for ARM consolidation in the MB. Here, we found that the outputs from both αβ and α′β′ neurons were required for complete ARM retrieval, suggesting two parallel ARM consolidation circuits additively contribute to retrieval. By RNAi-mediated knockdown experiments, the functional roles of *radish* and octopamine signaling in ARM consolidation were segregated in αβ and α′β′ neurons. Based on the segregation, we utilized the *rsh*^*1*^ mutant background to remove *radish*-dependent ARM that is consolidated in αβ neurons. In these *rsh*^*1*^ mutant flies, blocking output from αβ neurons during ARM retrieval failed to further impair the ARM expression, whereas blocking output from α′β′ neurons did, indicating the *radish*-independent ARM is mainly retrieved by the output of α′β′ neurons. We also showed that two MB output neurons downstream of αβ and α′β′ neurons, the MBON-β2β′2a and MBON-β′2mp neurons, respectively, mediated the ARM retrieval via glutamatergic transmission, supporting the model that two MB subsets, the αβ and α′β′ neurons, additively contribute to ARM consolidation and retrieval. Finally, we characterized the GAL4 lines expressing in all or subsets of MB α′β′ neurons [17], and functionally divided the α′β′ neurons into α′β′m neurons and α′β′ap neurons, whose outputs are required for ARM retrieval and ARM consolidation, respectively.

## Results

### Outputs from MB αβ and α′β′ neurons are required for 3-h ARM retrieval

**Fig 1:**
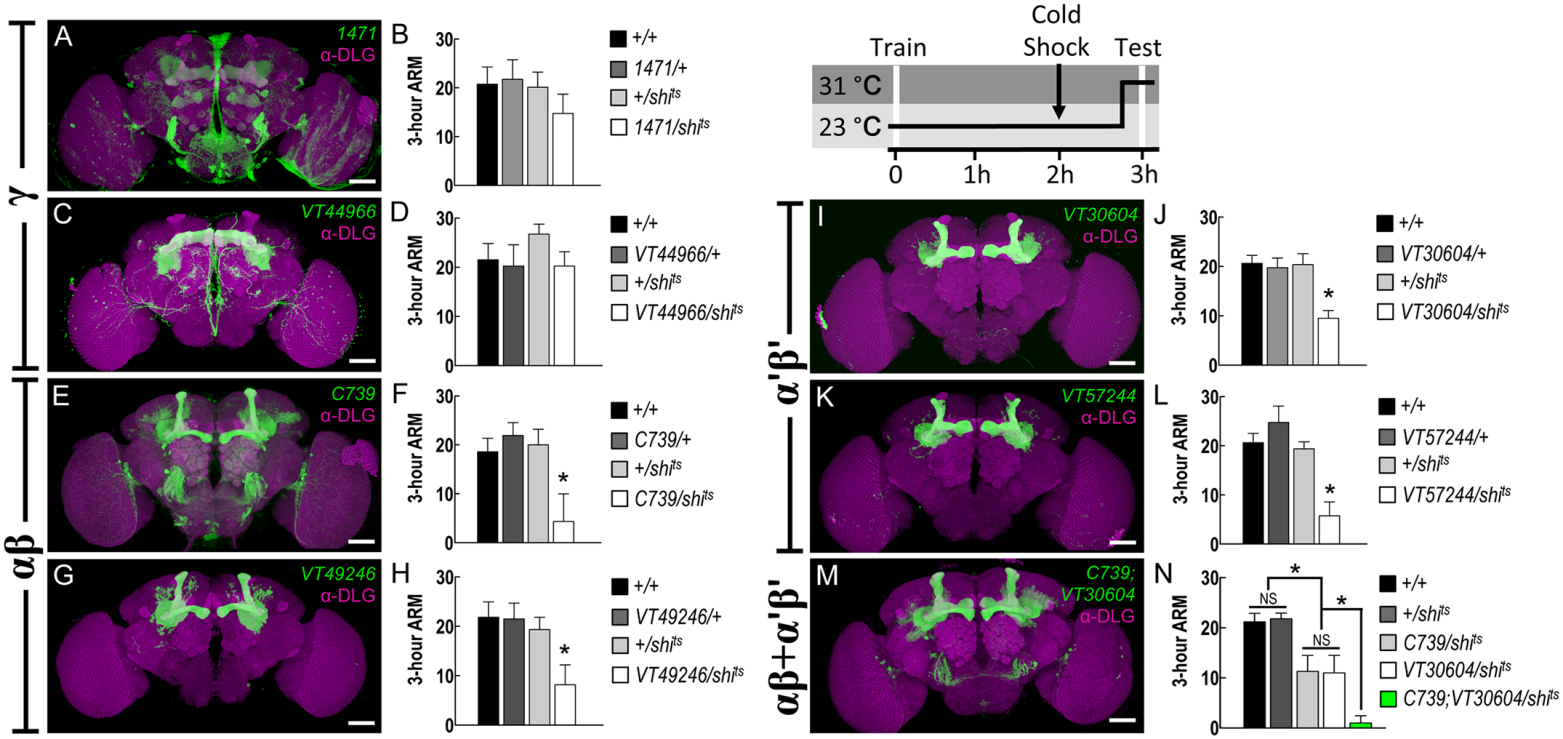
Outputs from MB αβ and α′β′ neurons are required for 3-h ARM retrieval. (A) Preferential expression of *1471-GAL4* in MB γ neurons (green). The brain was immunostained with DLG antibody (magenta). Scale bars represent 50 μm. Genotype was as follows: *1471-GAL4/UAS-mCD8*::*GFP; +/UAS-mCD8*::*GFP*. (B) Output from *1471-GAL4*-expressing neurons is not required for 3-h ARM retrieval. Each value represents mean ± SEM (*P* = 0.4394, N = 6 for each bar, ANOVA). Genotypes were as follows: (1) *+/+*, (2) *1471-GAL4/+; +/+*, (3) *+/+; +/UAS-shi*^*ts*^, (4) *1471-GAL4/+; +/UAS-shi*^*ts*^. (C) Preferential expression of *VT44966-GAL4* in MB γ neurons (green). The brain was immunostained with DLG antibody (magenta). Scale bars represent 50 μm. Genotype was as follows: +/*UAS-mCD8*::*GFP; VT44966-GAL4/UAS-mCD8*::*GFP*. (D) Output from *VT44966-GAL4*-expressing neurons is not required for 3-h ARM retrieval. Each value represents mean ± SEM (*P* = 0.3179, N = 8 for each bar, ANOVA). Genotypes were as follows: (1) *+/+*, (2) *+/+; VT44966-GAL4/+*, (3) *+/+; +/UAS-shi*^*ts*^, (4) *+/+; VT44966-GAL4/UAS-shi*^*ts*^. (E) Preferential expression of *C739-GAL4* in MB αβ neurons (green). The brain was immunostained with DLG antibody (magenta). Scale bars represent 50 μm. Genotype was as follows: *C739-GAL4/UAS-mCD8*::*GFP; +/UAS-mCD8*::*GFP*. (F) Output from *C739-GAL4*-expressing neurons is required for 3-h ARM retrieval. Each value represents mean ±SEM (*P = 0.0072, N = 12 for each bar, ANOVA followed by Tukey’s test). Genotypes were as follows: (1) *+/+*, (2) *C739-GAL4/+; +/+*, (3) *+/+; +/UAS-shi*^*ts*^, (4) *C739-GAL4/+; +/UAS-shi*^*ts*^. (G) Preferential expression of *VT49246-GAL4* in MB αβ neurons (green). The brain is immunostained with DLG antibody (magenta). Scale bars represent 50 μm. Genotype was as follows: +/*UAS-mCD8*::*GFP; VT49246-GAL4/UAS-mCD8*::*GFP*. (H) Output from *VT49246-GAL4*-expressing neurons is required for 3-h ARM retrieval. Each value represents mean ± SEM (*P = 0.0141, N = 12 for each bar, ANOVA followed by Tukey’s test). Genotypes were as follows: (1) *+/+*, (2) *+/+; VT49246-GAL4/+*, (3) *+/+; +/UAS-shi*^*ts*^, (4) *+/+; VT49246-GAL4/UAS-shi*^*ts*^. (I) Preferential expression of *VT30604-GAL4* in MB α′β′ neurons (green). The brain is immunostained with DLG antibody (magenta). Scale bars represent 50 μm. Genotype was as follows: +/*UAS-mCD8*::*GFP; VT30604-GAL4/UAS-mCD8*::*GFP*. (J) Output from *VT30604-GAL4*-expressing neurons is required for 3hr ARM retrieval. Each value represents mean ± SEM (*P = 0.0004, N = 8 for each bar, ANOVA followed by followed by Tukey’s test). Genotypes were as follows: (1) *+/+*, (2) *+/+; VT30604-GAL4/+*, (3) *+/+; +/UAS-shi*^*ts*^, (4) *+/+; VT30604-GAL4/UAS-shi*^*ts*^. (K) Preferential expression of *VT57244-GAL4* in MB α′β′ neurons (green). The brain is immunostained with DLG antibody (magenta). Scale bars represent 50μm. Genotype was as follows: +/*UAS-mCD8*::*GFP; VT57244-GAL4/UAS-mCD8*::*GFP*. (L) Output from *VT57244-GAL4*-expressing neurons is required for 3hr ARM retrieval. Each value represents mean ± SEM (*P < 0.0001, N = 8 for each bar, ANOVA followed by followed by Tukey’s test). Genotypes were as follows: (1) *+/+*, (2) *+/+; VT57244-GAL4/+*, (3) *+/+; +/UAS-shi*^*ts*^, (4) *+/+; VT57244-GAL4/UAS-shi*^*ts*^. (M) Preferential expression of *C739-GAL4;VT30604-GAL4* in MB αβ and α′β′ neurons (green). The brain is immunostained with DLG antibody (magenta). Scale bars represent 50μm. Genotype was as follows: *C739-GAL4*/*UAS-mCD8*::*GFP; VT30604-GAL4/UAS-mCD8*::*GFP*. (N) An additive effect of ARM deficiency in blocking synaptic transmissions from MB αβ and α′β′ neurons during memory retrieval. Each value represents mean ± SEM, N = 10 for each bar (*C739/shi*^*ts*^: *P = 0.0029 as compared to *+/+* and *+/shi*^*ts*^ groups, ANOVA followed by followed by Tukey’s test; *VT30604/shi*^*ts*^: *P = 0.0042 as compared to *+/+* and *+/shi*^*ts*^ groups, ANOVA followed by Tukey’s test; *C739;VT30604/shi*^*ts*^: *P = 0.0254 as compared to *C739/shi*^*ts*^ and *VT30604/shi*^*ts*^ groups, ANOVA followed by followed by Tukey’s test; NS, not significant: *P* = 0.7220 for *+/+* and *+/shi*^*ts*^, *P* = 0.9619 for *C739/shi*^*ts*^ and *VT30604/shi*^*ts*^, *t*-test). Genotypes were as follows: (1) *+/+*, (2)*+/+;* +/*UAS-shi*^*ts*^, (3) *C739-GAL4/+; +/UAS-shi*^*ts*^, (4) *+/+; VT30604-GAL4/UAS-shi*^*ts*^, (5) *C739-GAL4/+; VT30604-GAL4/UAS-shi*^*ts*^.

We first adopted a collection of GAL4 lines that represent specific subsets of MB neurons: *1471-GAL4* and *VT44966-GAL4* for γ neurons (Fig 1A and 1C); *C739-GAL4* and *VT49246-GAL4* for αβ neurons (Fig 1E and 1G); and *VT30604-GAL4* and *VT57244-GAL4* for α′β′ neurons (Fig 1I and 1K). We expressed the temperature-sensitive dominant-negative *dynamin* mutant transgene, *shibire* (*UAS-shi*^*ts*^) [23], using each of these GAL4 lines. Flies were trained to associate the electric shock and odor using classical conditioning protocol at 23°C, and a 2-min cold shock 2 hours after training was applied to assess 3-h ARM [4, 24]. We tested which MB subset output is required for 3-h ARM retrieval by shifting the temperature to 31°C 15 min prior to and during the test. Following previous studies that both αβ and γ neurons were required for 3-h memory retrieval [25, 26], we found that the output from αβ neurons is also required for 3-h ARM, a component of 3-h memory (Fig 1F and 1H) whereas the output from γ neurons is not (Fig 1B and 1D), suggesting that γ neurons might exclusively mediate retrieval of the other component, the ASM. This was confirmed by the observation that blocking the output from γ neurons using *VT44966-GAL4* impaired 3-h memory, instead of ARM, retrieval (S1 Fig). However, in contrast to the previous finding that α′β′ neurons are not required for 3-h memory retrieval [27], we found that blocking output from α′β′ neurons during retrieval using *VT30604-GAL4* or *VT57244-GAL4* impaired 3-h ARM (Fig 1J and 1L). This is a perplexing finding. Since 3-h ARM is a component of 3-h memory, any neural mechanism required for 3-h ARM should also be required for 3-h memory. This discrepancy prompted us to examine expression pattern of the GAL4 lines and eventually led to an explanation (see below).

We have used two independent GAL4 lines to reveal the functional role of αβ and α′β′ neurons in 3-h ARM retrieval. Next, we wonder whether there is an additive effect when blocking both MB subsets simultaneously. Although *VT49246-GAL4* line has more restricted expression pattern than *C739-GAL4* line, we only can use the double GAL4 line *C739-GAL4; VT30604-GAL4* expressing in both αβ and α′β′ neurons (Fig 1M) for *shibire* manipulation due to the genetic feasibility (see the materials and methods). Indeed, we found an additive effect in which 3-h ARM retrieval was reduced further in *C739-GAL4; VT30604-GAL4 > UAS-shi*^*ts*^ flies than in *C739-GAL4 > UAS-shi*^*ts*^ or *VT30604-GAL4 > UAS-shi*^*ts*^ flies (Fig 1N). This data indicated that outputs from αβ and α′β′ neurons additively contribute to 3-h ARM retrieval. All groups of flies showed no memory deficit at the permissive temperature and normal avoidance of odor or shock at the restrictive temperature (S2 Fig).

### RADISH in MB αβ neurons and octopamine signaling in α′β′ neurons mediate 3-h ARM consolidation

**Fig 2:**
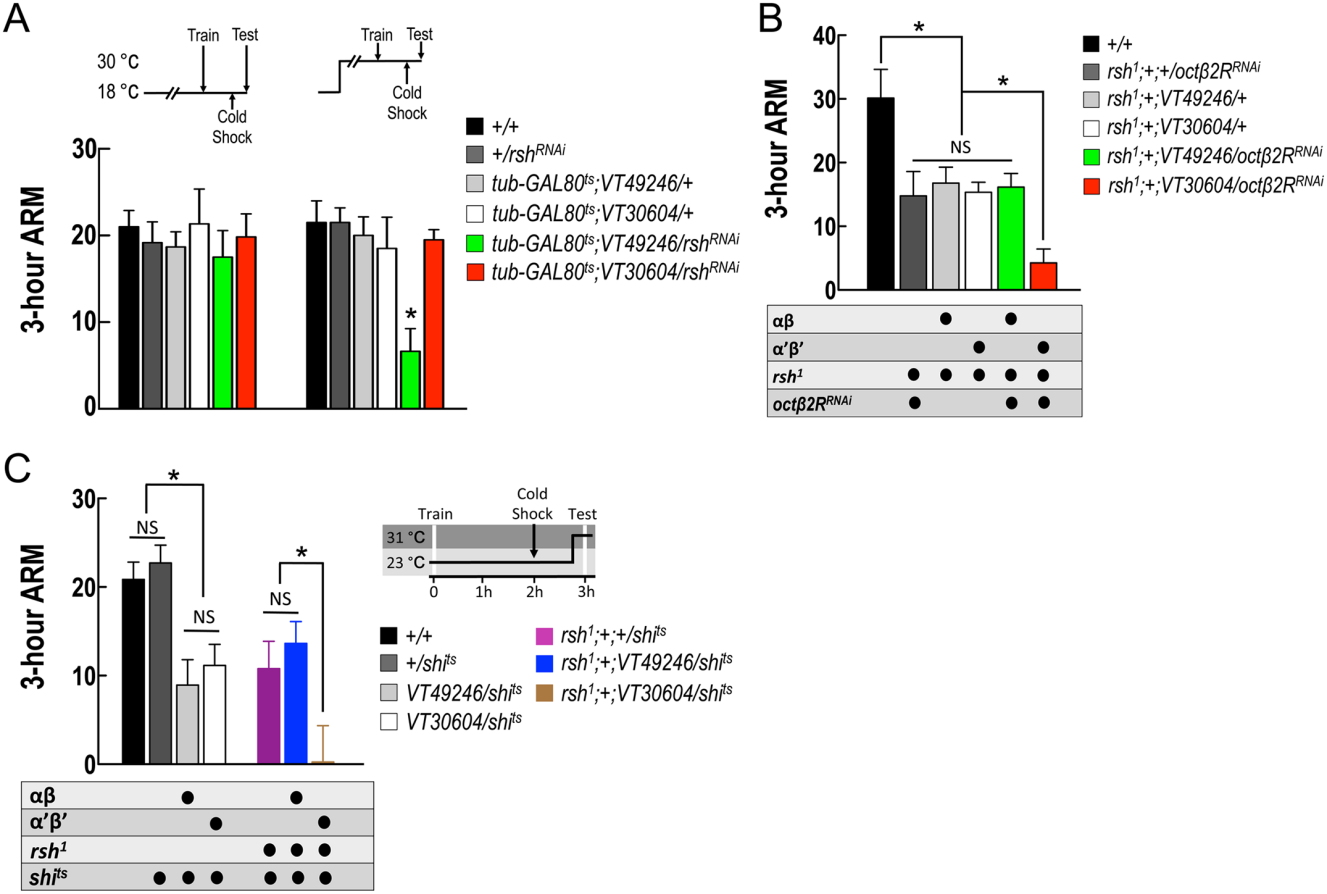
RADISH in αβ neurons and octopamine signaling in α′β′ neurons additively contribute to 3-h ARM consolidation. (A) Adult-stage-specific knockdown of *radish* in MB αβ but not α′β′ neurons impaired ARM. Each value represents mean ± SEM (left panel: *P* = 0.9199, N = 6, ANOVA; right panel: *P = 0.0005, N = 8, ANOVA followed by followed by Tukey’s test). Genotypes were as follows: (1) *+/+*, (2) *+/UAS-radish*^*RNAi*^*(v39931); +/+*, (3) *tub-GAL80*^*ts*^*/+; VT49246-GAL4/+*, (4) *tub-GAL80*^*ts*^*/+; VT30604-GAL4/+*, (5) *tub-GAL80*^*ts*^*/UAS-radish*^*RNAi*^*(v39931); VT49246-GAL4/+*, (6) *tub-GAL80*^*ts*^*/UAS-radish*^*RNAi*^*(v39931); VT30604-GAL4/+*. (B) In *rsh*^*1*^ background, knockdown of *octβ2R* in MB α′β′ but not αβ neurons further impaired 3-h ARM. In all tests, ASM was removed by cold-induced anesthesia. Each value represents mean ± SEM (*rsh*^*1*^*; +; VT30604/octβ2R*^*RNAi*^: *P = 0.0075 as compared to all *rsh*^*1*^ background controls, N = 8 for each bar, ANOVA followed by followed by Tukey’s test). Genotypes were as follows: (1)*+/+*, (2) *rsh*^*1*^*; +/+; +/UAS-octβ2R*^*RNAi*^*(v104524)*, (3) *rsh*^*1*^*; +/+; VT49246-GAL4/+*, (4) *rsh*^*1*^*; +/+; VT30604-GAL4/+*, (5) *rsh*^*1*^*; +/+; VT49246-GAL4/UAS-octβ2R*^*RNAi*^*(v104524)*, (6) *rsh*^*1*^*; +/+; VT30604-GAL4/UAS-octβ2R*^*RNAi*^*(v104524)*. (C) In *radish* mutant (*rsh*^*1*^) background, blocking neurotransmission from MB α′β′ but not αβ neurons during memory retrieval further impaired 3-h ARM. Each value represents mean ± SEM (*VT49246/shi*^*ts*^: *P = 0.0002 as compared to *+/+* and *+/shi*^*ts*^ controls, N = 14 for each bar, ANOVA followed by followed by Tukey’s test; *VT30604/shi*^*ts*^: *P = 0.0008 as compared to *+/+* and *+/shi*^*ts*^ controls, N = 14 for each bar, ANOVA followed by Tukey’s test; *rsh*^*1*^*;+;VT30604/shi*^*ts*^: *P = 0.0124 as compared to *rsh*^*1*^*;+;+/shi*^*ts*^ and *rsh*^*1*^*;+;VT49246/shi*^*ts*^, N = 16 for each bar, ANOVA followed by Tukey’s test). Genotypes were as follows: (1) *+/+*, (2) *+/+; +/UAS-shi*^*ts*^, (3) *+/+; VT49246-GAL4/UAS-shi*^*ts*^, (4) *+/+; VT30604-GAL4/UAS-shi*^*ts*^, (5) *rsh*^*1*^*; +/+; +/UAS-shi*^*ts*^, (6) *rsh*^*1*^*; +/+; VT49246-GAL4/UAS-shi*^*ts*^, (7) *rsh*^*1*^*; +/+; VT30604-GAL4/UAS-shi*^*ts*^

The *radish* gene encodes a protein that is required for ARM formation and is preferentially immunolabeled in the α, β, and γ lobes as well as the calyx and ellipsoid body [19]. A previous study showed that feeding *rsh*^*1*^ mutant flies with serotonin synthesis inhibitor had no effect on 3-h memory, suggesting that RADISH and serotonergic DPM–αβ neurons circuit are in the same pathway for ARM consolidation [21]. Also, our previous study showed an additive effect on ARM deficit when combining knockdown of octopamine synthesis gene in APL neurons with serotonin synthesis inhibitor or with *rsh*^*1*^ mutant background, suggesting that octopaminergic APL–α′β′ neurons and *radish*-dependent serotonergic DPM–αβ neurons circuits independently mediate ARM consolidation [22]. However, all the *radish*-related studies are mainly based on *rsh*^*1*^ mutant background, and the subset of MB neurons in which *radish* functions for ARM consolidation has not been identified. We used an inducible RNAi-mediated knockdown strategy to suppress *radish* expression in the adult stage. Flies with the *tubulin* promoter-driven temperature-sensitive GAL4 repressor GAL80 (*tub-GAL80*^*ts*^) were raised at 18°C and transferred to 30°C for 7 days after eclosion. Inducible RNAi-mediated knockdown of *radish* (see S3A and S3B Fig for validation by quantitative PCR) in αβ neurons using *VT49246-GAL4*, but not in α′β′ neurons using *VT30604-GAL4*, caused a significant deficit (Fig 2A), suggesting that RADISH mediates ARM consolidation in αβ neurons. Normal *radish* expression in γ neurons is not required for 3-h ARM (S3C Fig). We also used a combinatorial assay to confirm that the octopamine signaling for ARM consolidation in α′β′ neurons is independent of *radish*. In the *rsh*^*1*^ mutant background, RNAi-mediated knockdown of *octβ2R* in α′β′ neurons, but not in αβ neurons, further impaired ARM (Fig 2B). Taken together, these data showed that RADISH in αβ neurons and octopamine signaling in α′β′ neurons mediated ARM consolidation in parallel.

### Output from α′β′ neurons mediates *radish*-independent ARM retrieval

To further demonstrate the independency between two parallel neural pathways/circuits expressing ARM, we conducted an experiment in which gene for memory consolidation and neurotransmission for retrieval were manipulated in the same flies. We first confirmed that blocking neurotransmission during retrieval in either αβ neurons using *VT49246-GAL4* or α′β′ neurons using *VT30604-GAL4* impaired ARM expression (Fig 2C, left panel). After switching the genetic background to *rsh*^*1*^ to disrupt *radish*-dependent ARM consolidation, only neurotransmission blockade in α′β′ neurons, but not in αβ neurons, during retrieval caused further reduction of ARM expression (Fig 2C, right panel). All *shibire*-expressing flies in *rsh*^*1*^ background showed normal avoidance of odor or shock at the restrictive temperature (S3D Fig). These data indicate that the output from α′β′ neurons mediates *radish*-independent ARM retrieval while the output from αβ neurons, not surprisingly, mediates *radish*-dependent ARM retrieval.

### Glutamatergic MB output neurons downstream of αβ and α′β′ neurons are required for 3-h ARM retrieval

**Fig 3:**
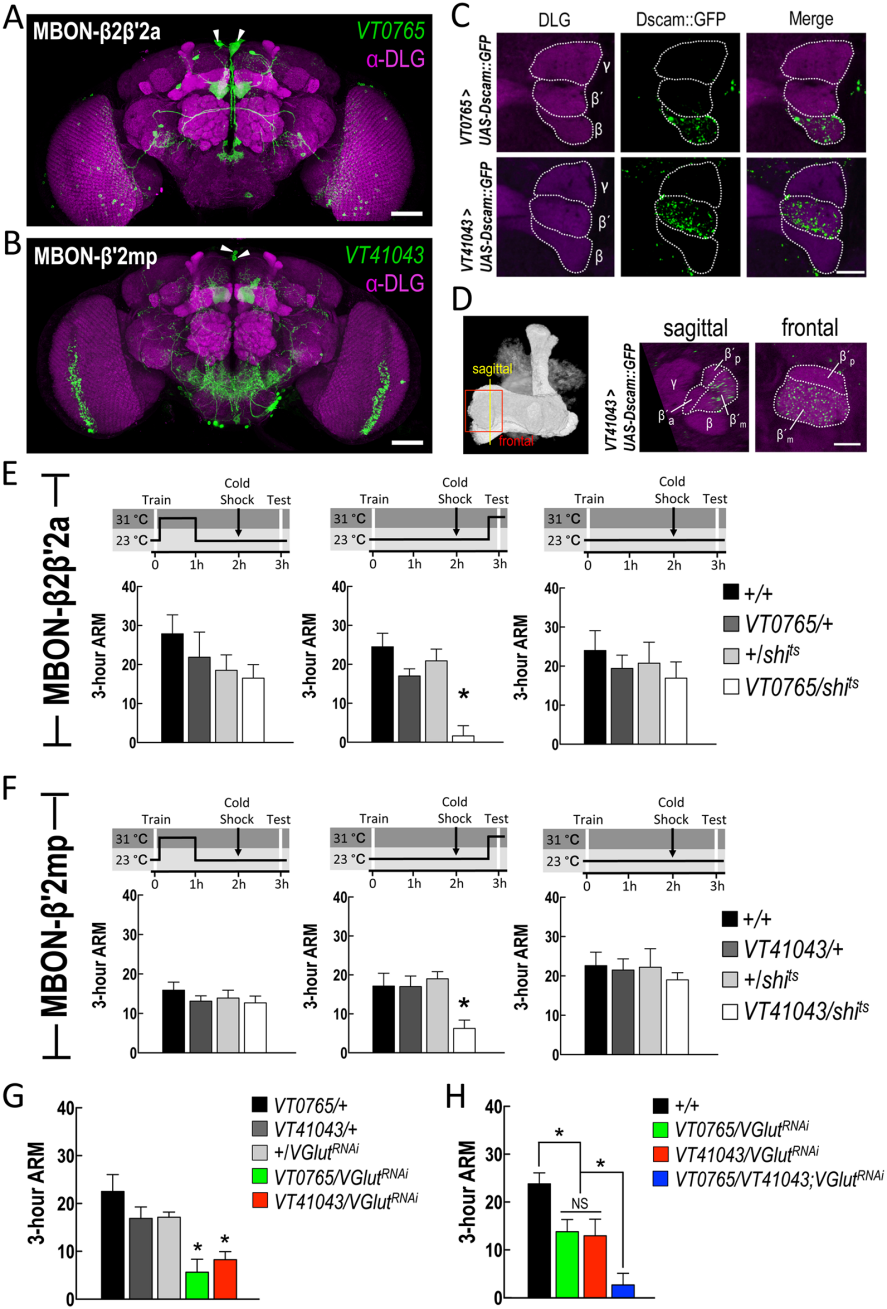
Glutamatergic MB αβ and α′β′ output neurons are required for 3-h ARM retrieval. (A) The expression pattern of *VT0765-GAL4* (green) which specifically labels MBON-β2β′2a neurons. The brain was immunostained with DLG antibody (magenta). Arrowheads indicate the somata of MBON-β2β′2a neurons. The scale bar represents 50 μm. Genotype was as follows: *+/UAS-mCD8*::*GFP; VT0765-GAL4/UAS-mCD8*::*GFP*. (B) The expression pattern of *VT41043-GAL4* (green) which specifically labels the MBON-β′2mp neurons. The brain is immunostained with DLG antibody (magenta). Arrowheads indicate the somata of MBON-β′2mp neurons. The scale bar represents 50 μm. Genotype was as follows: *+/UAS-mCD8*::*GFP; VT41043-GAL4/UAS-mCD8*::*GFP*. (C) Sub-regional dendritic distributions of MBON-β2β′2a and MBON-β′2mp neurons with *Dscam*::*GFP* positive signals (green). Brains were counterstained with DLG antibody (magenta). The scale bar represents 10 μm. Genotypes: (1) *+/UAS-Dscam[17*.*1]*::*GFP; +/+; VT0765-GAL4/+*, (2) *+/UAS-Dscam[17*.*1]*::*GFP; +/+; VT41043-GAL4/+*. (D) Sagittal and frontal views of dendritic distributions of MBON-β′2mp neurons. The left panel indicates the regions selected for analyses. The brain was counterstained with DLG antibody (magenta). The scale bar represents 10 μm. Genotype: +/*UAS-Dscam[17*.*1]*::*GFP; +/+; VT41043-GAL4/+*. (E) Neurotransmission from MBON-β2β′2a neurons is required for retrieval but not consolidation of 3-h ARM. Each value represents mean ± SEM (left panel: *P* = 0.3772, N = 8, ANOVA; middle panel: *P < 0.0001, N = 8, ANOVA followed by Tukey’s test; right panel: *P* = 0.7412, N = 12, ANOVA). Genotypes were as follows: (1) *+/+*, (2) *+/+; VT0765-GAL4/+*, (3) *+/+; +/UAS-shi*^*ts*^, (4) *+/+; VT0765-GAL4/UAS-shi*^*ts*^. (F) Neurotransmission from MBON-β′2mp neurons is required for retrieval but not consolidation of 3-h ARM. Each value represents mean ± SEM (left panel: *P* = 0.6024, N = 9 for each bar, ANOVA; middle panel: *P = 0.0058, N = 8, ANOVA followed by Tukey’s test; right panel: *P* = 0.8746, N = 10 for each bar, ANOVA). Genotypes were as follows: (1) *+/+*, (2) *+/+; VT41043-GAL4 /+*, (3) *+/+; +/UAS-shi*^*ts*^, (4) *+/+; VT41043-GAL4/UAS-shi*^*ts*^. (G) Knockdown of *VGlut* in MBON-β2β′2a or MBON-β′2mp neurons impaired 3-h ARM. Each value represents mean ± SEM, N = 8 for each bar (*VT0765/VGlut*^*RNAi*^: *P = 0.0007 as compared to *VT0765/+* and *+/VGlu*^*RNAi*^ controls, ANOVA followed by Tukey’s test; *VT41043/VGlut*^*RNAi*^: *P = 0.0022, as compared to *VT41043/+* and *+/VGlu*^*RNAi*^ controls, ANOVA followed by Tukey’s test). Genotypes were as follows: (1) *+/+; VT0765-GAL4/+*, (2) *+/+; VT41043-GAL4/+*, (3) *+/UAS-VGlut*^*RNAi*^*(v104324); +/+*, (4) *+/UAS-VGlut*^*RNAi*^*(v104324); VT0756-GAL4/+*, (5) *+/UAS-VGlut*^*RNAi*^*(v104324); VT41043-GAL4/+*. (H) An additive effect of ARM deficiency in knockdown of *VGlut* in MBON-β2β′2a and MBON-β′2mp neurons. Each value represents mean ± SEM, N = 14 for each bar (*VT0765/VT41043; VGlut*^*RNAi*^: *P = 0.0148 as compared to *VT0765/VGlut*^*RNAi*^ and *VT41043/VGlu*^*RNAi*^ controls, ANOVA followed by Tukey’s test; NS: not significant). Genotypes were as follows: (1) *+/+*, (2) *+/UAS-VGlut*^*RNAi*^*(v104324); VT0765-GAL4/+*, (3) *+/UAS-VGlut*^*RNAi*^*(v104324); VT41043-GAL4/+*, (4) *+/UAS-VGlut*^*RNAi*^*(v104324); VT0765-GAL4/VT41043-GAL4*.

MBON-β2β′2a and MBON-β′2mp neurons are two pairs of MB output neurons for αβ and α′β′ neurons, respectively [17, 28, 29] (see also Fig 3A and 3B). The dendrites of the MBON-β2β′2a neuron were marked by *Dscam*::*GFP* in the β lobe tips, whereas the dendrites of the MBON-β′2mp neuron were found only in the middle stratum of the β′ lobe (Fig 3C and 3D), albeit sparse *DenMark*-positive signals were additionally seen in the β′2a region for the MBON-β2β′2a neuron (S4A Fig)[17]. Blocking neurotransmission from MBON-β2β′2a or MBON-β′2mp neurons during the test, but not the first hour after training, impaired ARM expression (Fig 3E and 3F), indicating that the outputs from these neurons are required for ARM retrieval. All *shibire*-expressing flies showed normal avoidance of odor or shock at the restrictive temperature (S4B Fig). It has been shown that both MBON-β2β′2a and MBON-β′2mp neurons are vesicular glutamate transporter- (VGlut-) antibody immunopositive, which is indicative of glutamatergic neuron in *Drosophila melanogaster* [28,30]. To determine whether glutamatergic transmission mediates the retrieval, we knocked down *VGlut* expression in MBON-β2β′2a or MBON-β′2mp neurons by RNAi (see S5A Fig for validation of *UAS-VGlut*^*RNAi*^ by quantitative PCR) and tested the flies for 3-h ARM. *VT0765-GAL4 > UAS-VGlut*^*RNAi*^ and *VT41043-GAL4* > *UAS-VGlut*^*RNAi*^ flies showed 3-h ARM deficit (Fig 3G). Furthermore, knockdown of *VGlut* in both MBON-β2β′2a and MBON-β′2mp neurons showed an additive effect on ARM deficit (Fig 3H). Consistent with the additive contributions of αβ and α′β′ neurons to 3-h ARM retrieval, this additive effect suggests that glutamatergic MBON-β2β′2a and MBON-β′2mp neurons also additively contribute to ARM retrieval, although blocking output from the former alone was sufficient to abolish 3-h ARM. Since these experiments adopted chronic knockdown of *VGlut* that may provoke secondary effects, we introduced *tub-GAL80*^*ts*^ for inducible *VGlut* knockdown in the adult stage (S5B Fig), confirming that losing *VGlut* itself in MBON-β2β′2a or MBON-β′2mp neurons impaired ARM. In summary, our data draw two sets of double-layered parallel circuits additively expressing 3-h ARM in the fruit fly.

### Characterization of GAL4 lines expressing in α′β′ neurons

**Fig 4:**
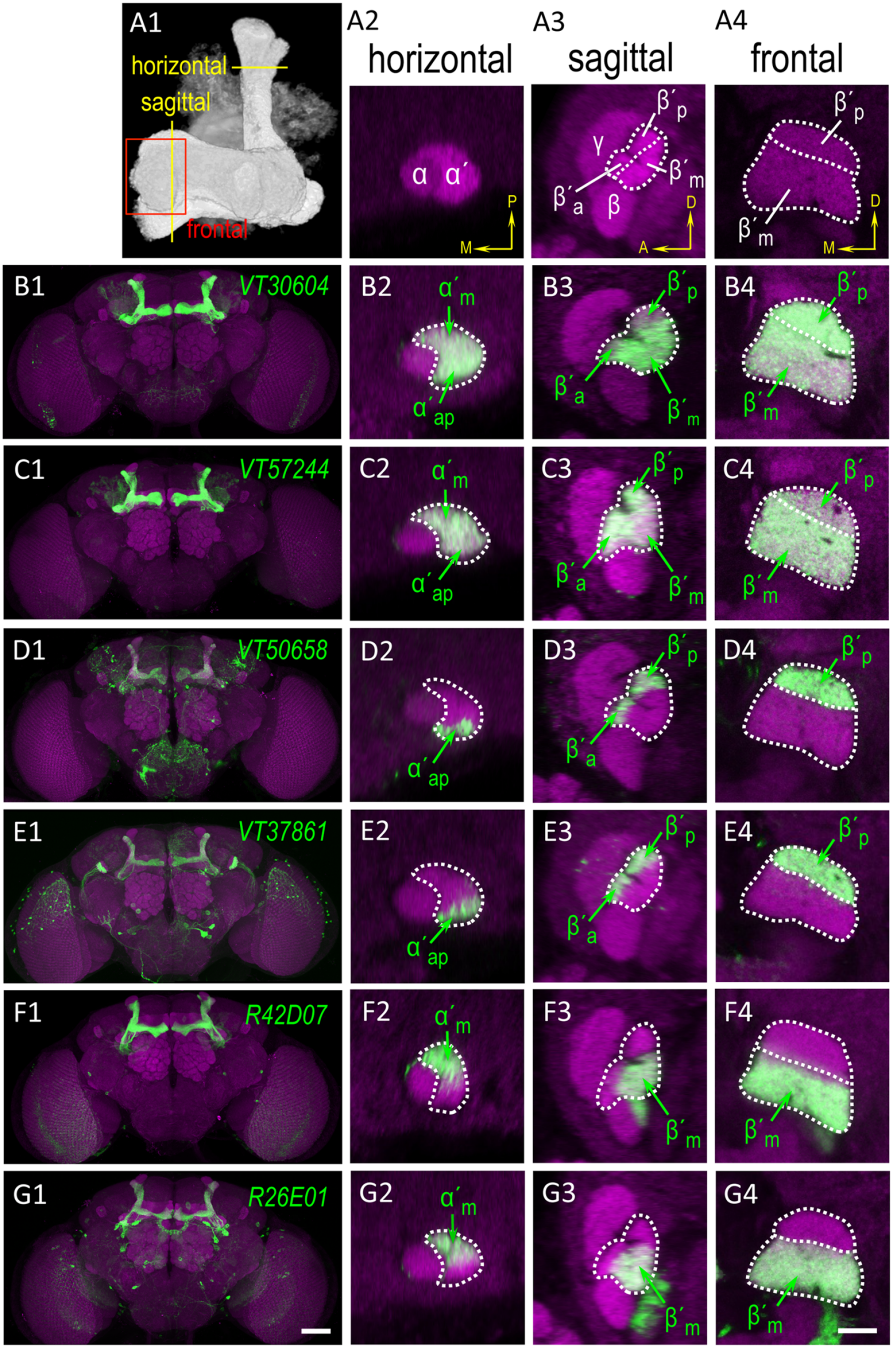
Characterization of GAL4 lines expressing in α′β′ neurons. (A1–A4) The MB structure indicating the regions selected for analyses (A1), and high-magnification single horizontal (A2), sagittal (A3), and frontal (A4) confocal cross sections of the MB lobes counterstained with DLG antibody (magenta). Yellow arrows indicate the orientation of the brain: M, medial; P, posterior; A, anterior; D, dorsal. (B1–B4) Preferential expression of *VT30604-GAL4* in MB α′β′ap and α′β′m neurons (green). Genotype was as follows: +/*UAS-mCD8*::*GFP; VT30604-GAL4/UAS-mCD8*::*GFP*. (C1–C4) Preferential expression of *VT57244-GAL4* in MB α′β′ap and α′β′m neurons (green). Genotype was as follows: +/*UAS-mCD8*::*GFP; VT57244-GAL4/UAS-mCD8*::*GFP*. (D1–D4) Preferential expression of *VT50658-GAL4* in MB α′β′ap neurons (green). Genotype was as follows: *+/UAS-mCD8*::*GFP; VT50658-GAL4/UAS-mCD8*::*GFP*. (E1–E4) Preferential expression of *VT37861-GAL4* in MB α′β′ap neurons (green). Genotype was as follows: *+/UAS-mCD8*::*GFP; VT37861-GAL4/UAS-mCD8*::*GFP*. (F1–F4) Preferential expression of *R42D07-GAL4* in MB α′β′m neurons (green). Genotype was as follows: *+/UAS-mCD8*::*GFP; R42D07-GAL4/UAS-mCD8*::*GFP*. (G1–G4) Preferential expression of *R26E01-GAL4* in MB α′β′m neurons (green). Genotype was as follows: *+/UAS-mCD8*::*GFP; R26E01-GAL4/UAS-mCD8*::*GFP*. The scale bars represent 50 μm in G1 and 10 μm in G4.

Krashes et al. used *C305a-GAL4* and *C320-GAL4* lines to conclude that the output from α′β′ neurons was required for 3-h memory acquisition and consolidation but not for 3-h memory retrieval [27](see also S6D1–S6D2 and S6E1–S6E2 Fig). Since 3-h memory can be dissected into ASM and ARM, a neural mechanism required for 3-h ARM should intuitively be required for 3-h memory. However, the output from α′β′ neurons was shown to be required for the retrieval of 3-h ARM in our study (Fig 1J and 1L), arguing that the output from α′β′ neurons should be required for the retrieval of 3-h memory. In order to reconcile this conflict, we closely revisited the GAL4 expression pattern and found that although *C305a-GAL4* expresses in both α′β′ap and α′β′m subsets, the marked GFP signal did not occupied the whole region of each stratum of α′/β′ lobes in magnified horizontal, sagittal, and frontal sections (S6B1–S6B4 Fig), especially the middle stratum. The other more restricted *C320-GAL4* expresses mainly in α′β′ap neurons and has no noticeable GFP signal in the middle stratum, either (S6C1–S6C4 Fig). In contrast, *VT30604-GAL4* and *VT57244-GAL4* expression patterns occupied strongly and comprehensively all strata of the α′/β′ lobes (Fig 4B1–4B4 and 4C1–4C4), suggesting that these two VT lines express in most if not all α′β′ neurons, while *C305a-GAL4* and *C320-GAL4* express weakly in a subpopulation that has few α′β′m subset neurons. Consistently, cell-counting data showed that *VT30604-GAL4* or *VT57244-GAL4* expresses in about twice as many MB neurons as *C305a-GAL4* or *C320-GAL4* does (Table 1). This conclusion derived from imaging observation led to a speculation that using *C305a-GAL4* or *C320-GAL4* for *shibire* manipulation, which requires high enough expression level to perturb synaptic transmission [23], cannot reflect the full functional role of α′β′ neurons, instead a skewed role for α′β′ap subset. Hence, we sought specific GAL4 lines expressing in the subsets of α′β′ neurons to decisively address this issue. By visually screening the Vienna Tile (VT) library and Janelia collection, we identified the *VT50658-GAL4* and *VT37861-GAL4* lines for α′β′ap neurons (Fig 4D1–4D4 and 4E1–4E4) as well as the *R42D07-GAL4* and *R26E01-GAL4* lines for α′β′m neurons (Fig 4F1–4F4 and 4G1–4G4). Take counts of MB neurons labeled by these GAL4 lines into consideration (Table 1), we decided *VT30604-GAL4*, *VT37861-GAL4*, and *R42D07-GAL4* as good single-transgene GAL4 lines to study α′β′, α′β′ap, and α′β′m neurons, respectively.

**Table 1:** The numbers of genetically labeled MB neurons in different GAL4 lines expressing in α′β′ neurons.

GAL4 driver	Number of labeled MB neurons per hemisphere (mean ± SEM)
***C305a***	362.00±13.47
***C320***	383.75±14.78
***VT30604***	725.10±15.53
***VT57244***	900.00±22.14
***VT50658***	183.38±6.56
***VT37861***	376.25±13.77
***R42D07***	405.25±7.77
***R26E01***	342.75±4.05

The numbers of labeled MB neurons were counted from 5-day old flies for both genders. Values represent mean ± SEM (N = 8 for each number).

### Different roles of α′β′ap and α′β′m neurons in ARM

**Fig 5:**
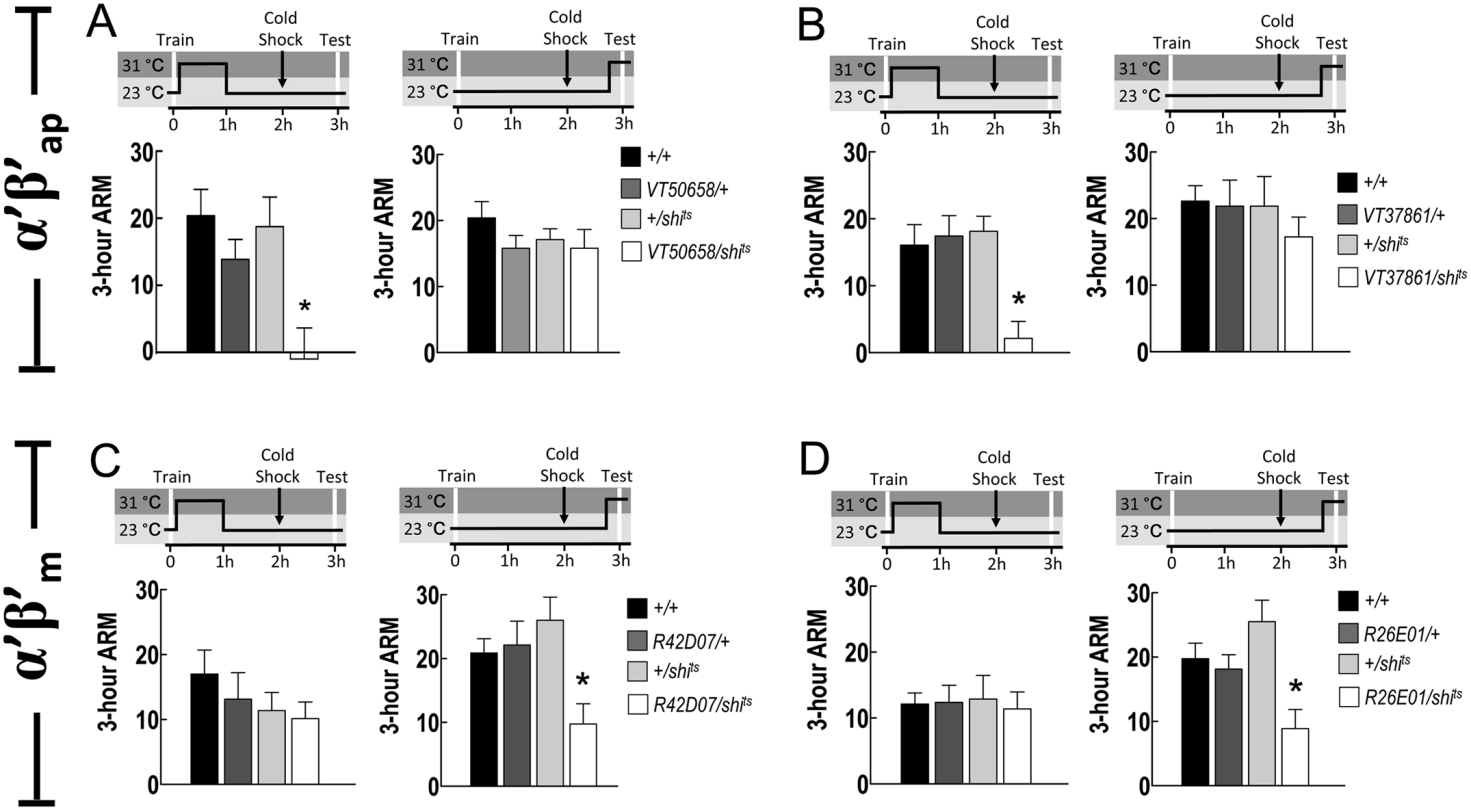
Output from MB α′β′ap is required for 3-h ARM consolidation while output from α′β′m neurons for retrieval. (A) Output from *VT50658-GAL4* expression neurons is required for consolidation but not retrieval of 3-h ARM. Each value represents mean ± SEM (left panel: *P = 0.0033, N = 8 for each bar, ANOVA followed by Tukey’s test; right panel: *P* = 0.4413, N = 10 for each bar, ANOVA). Genotypes were as follows: (1) *+/+*, (2) *+/+; VT50658-GAL4/+*, (3) *+/+; +/UAS-shi*^*ts*^, (4) *+/+; VT50658-GAL4/UAS-shi*^*ts*^. (B) Output from *VT37861-GAL4* expression neurons is required for consolidation but not retrieval of 3-h ARM. Each value represents mean ± SEM (left panel: *P = 0.0002, N = 14 for each bar, ANOVA followed by Tukey’s test; right panel: *P* = 0.6916, N = 8 for each bar, ANOVA). Genotypes were as follows: (1) *+/+*, (2) *+/+; VT37861-GAL4/+*, (3) *+/+; +/UAS-shi*^*ts*^, (4) *+/+; VT37861-GAL4/UAS-shi*^*ts*^. (C) Output from *R42D07-GAL4* expression neurons is required for retrieval but not consolidation of 3-h ARM. Each value represents mean ± SEM (left panel: *P* = 0.5023, N = 8 for each bar, ANOVA; right panel: *P = 0.0094, N = 8 for each bar, ANOVA followed by Tukey’s test). Genotypes were as follows: (1) *+/+*, (2) *+/+; R42D07-GAL4/+*, (3) *+/+; +/UAS-shi*^*ts*^, (4) *+/+; R42D07-GAL4/UAS-shi*^*ts*^. (D) Output from *R26E01-GAL4* expression neurons is required for retrieval but not consolidation of 3-h ARM. Each value represents mean ± SEM (left panel: *P* = 0.9823, N = 8 for each bar, ANOVA; right panel: *P = 0.0024, N = 8 for each bar, ANOVA followed by Tukey’s test). Genotypes were as follows: (1) *+/+*, (2) *+/+; R26E01-GAL4/+*, (3) *+/+; +/UAS-shi*^*ts*^, (4) *+/+; R26E01-GAL4/UAS-shi*^*ts*^.

To clarify the functional roles of different subsets of MB α′β′ neurons in ARM, we used the GAL4 lines characterized above for *shibire* manipulation. Blocking the output from α′β′ap neurons using *VT50658-GAL4* or *VT37861-GAL4* during the first hour after training impaired subsequent ARM expression whereas the same manipulation during retrieval had no effect (Fig 5A and 5B), suggesting that output from the α′β′ap neurons is involved in ARM consolidation. This involvement in 3-h ARM consolidation, but not retrieval, is an analogy to the finding with the *C305a-GAL4* and *C320-GAL4* lines, in which output from GAL4-expressing neurons is required for 3-h memory consolidation but not retrieval [27] (see also S6 Fig). In contrast, blocking the output from α′β′m neurons using *R42D07-GAL4* or *R26E01-GAL4* during retrieval impaired ARM expression whereas the same manipulation during the first hour after training had no effect (Fig 5C and 5D), suggesting that the output from α′β′m neurons mediates 3-h ARM retrieval, despite the concern that the overall low scores make us hesitate to exclude the involvement in consolidation (left panels of Fig 5C and 5D). This role of α′β′m neurons is also supported by the dendritic distribution of the MBON-β′2mp neurons, which exclusively occupied the middle stratum of the β′ lobe (Fig 3D). Given that these flies showed normal avoidance of odor or shock at restrictive temperature (S7A1–S7A4 and S7B Fig), the data collectively indicate the functional heterogeneity of MB α′β′ neurons, where outputs from the α′β′ap and α′β′m neurons mediate the ARM consolidation and retrieval, respectively.

## Discussion

The key finding in our study is the identification of two parallel neural pathways that additively express 3-h aversive ARM through *Drosophila* MB αβ and α′β′ neurons. After training, RADISH in MB αβ neurons and octopamine signaling in α′β′ neurons independently consolidate ARM, which is additively retrieved by αβ–MBON-β2β′2a and α′β′m–MBON-β′2mp circuits for memory expression. Five lines of evidence support this scenario. First, the output from αβ or α′β′ neurons is required for ARM retrieval (Fig 1F, 1H, 1J and 1L), and the effect of blocking αβ output and that of blocking α′β′ output during retrieval are additive (Fig 1N). Second, knockdown of *radish* in αβ neurons, but not in α′β′ neurons, impaired ARM (Fig 2A), while knockdown of *octβ2R* in α′β′ neurons further impaired the residual ARM in *rsh*^*1*^ mutant flies (Fig 2B). Third, blocking output from α′β′ neurons, but not from αβ neurons, during retrieval further impaired the residual ARM in *rsh*^*1*^ mutant flies (Fig 2C). Forth, glutamatergic output from neurons downstream of the αβ or α′β′ neurons, i.e., MBON-β2β′2a or MBON-β′2mp neurons, is required for ARM retrieval, and the effects of knockdown of *VGlut* are additive (Fig 3). Finally, output from α′β′m neurons, but not α′β′ap neurons, is required for ARM retrieval, consistent with the dendritic distribution of MBON-β′2mp neurons (Figs 3D and 5).

The parallel pathways for 3-h ARM expression were spatially defined by the requirements of neurotransmission from two sets of circuits during retrieval, the αβ–MBON-β2β′2a neurons and the α′β′m–MBON-β′2mp neurons. In addition, blocking neurotransmission from αβ or α′β′ neurons during retrieval reduced ARM expression by about 50% (Fig 1H, 1J and 1L) whereas simultaneous blockade produced an additive effect that completely abolished ARM expression (Fig 1N). Similar additive effects were repeatedly observed in experiments that utilize manipulations in both pathways: an *rsh*^*1*^ mutant background plus *octβ2R* RNAi knockdown (Fig 2B) or plus retrieval blockade in α′β′ neurons (Fig 2C), and knockdown of *VGlut* in MBON-β2β′2a plus MBON-β′2mp neurons (Fig 3H). Thus, total four lines of evidence support the additive expression of 3-h ARM.

The parallel pathways for 3-h ARM expression shown here differ from the degenerate parallel pathways for the stomatogastric ganglion of the crab or CO_2_ avoidance in the fly [31, 32], as the latter enable mechanisms by which the network output can be switched between states. In our study, the two parallel neural pathways additively contribute to the expression of 3-h ARM. The nature of the ARM parallel pathways may be similar to that for cold avoidance behavior in the fly, where parallel pathways in the β′ and β circuits additively contribute but only the β circuit allows age-dependent alterations for potential benefits against aging [29]. Considering the robustness of ARM through the course of senescence [5], it’s unlikely to be age-dependent alterations in ARM system.

In studies of *Drosophila* neurobiology, *C305a-GAL4* is a common GAL4 line for α′β′ neurons [16, 27]. Here, by examining three different zoom-in sections of the MB lobes and counting the cells (S6B1–S6B4 Fig and Table 1), we extensively characterized the following GAL4 lines expressing in α′β′ neurons: *VT30604-GAL4* and *VT57244-GAL4*, which cover most α′β′ap and α′β′m neurons; *VT37861-GAL4* and *VT50658-GAL4*, which cover α′β′ap neurons; and *R42D07-GAL4* and *R26E01-GAL4*, which cover most α′β′m neurons. In contrast, *C305a-GAL4* sporadically expresses in about half as many MB neurons as *VT30604-GAL4* or *VT57244-GAL4* does (Table 1). Although covering both subsets of α′β′ neurons, the expression pattern of *C305a-GAL4* in α′β′m neurons is too few and/or weak to lead to a perturbation of synaptic transmission. This is shown by the data that retrieval of 3-h ARM was disrupted by *shibire* manipulation using all-α′β′ neurons driver (Fig 1J and 1L) or α′β′m-specific driver (right panels of Fig 5C and 5D), but neither α′β′ap-specific driver (right panels of Fig 5A and 5B) nor *C305a-GAL4* for 3-h memory ([27], see also S6D2 Fig). Please note that our GFP signals were acquired from flies carrying two copies of *5XUAS-mCD8*::*GFP* reporter and without any immunostaining-mediated amplification. With the assistance of immunostaining and/or advanced reporter such as increasing copy number of UAS or incorporating a small intron to boost expression [33], some studies have shown appreciable GFP signal in most α′β′ neurons [16, 27, 34]. Given that *shibire*-mediated neurotransmission blockade and RNAi-mediated knockdown require high enough expression level, the imaging method we adopted in this study can faithfully reflect the regions that were effectively manipulated in our, as well as Krashes et al’s [27], behavioral assays. Regarding the pervasive use of *C305a-GAL4* for *shibire* or RNAi manipulation, some functional studies of α′β′ neurons might need to be carefully revisited. Here, we showed, by close examination and cell counting, *VT30604-GAL4*, *VT37861-GAL4*, and *R42D07-GAL4* as useful GAL4 lines to study α′β′, α′β′ap, and α′β′m neurons, respectively, especially when split-GAL4 lines that span the second and third chromosomes are not genetically feasible [17].

ARM was thought to be diminished in *radish* mutant flies, in which a truncated RADISH is expressed [19]. It’s noteworthy that *radish* mutants still show a residual 3-h ARM with a PI of roughly 10, which is equal to the 3-h ARM score in wild-type flies fed with an inhibitor of serotonin synthesis to hinder the serotonergic DPM neurotransmission [19, 21, 22]. Interestingly, feeding *radish* mutant flies with the drug didn’t make the 3-h memory score worse [21], which has already implied that RADISH mediates the consolidation of ARM in the serotonergic DPM-αβ neurons circuit. Indeed, in this study we took advantage of RNAi-mediated knockdown to identify αβ neurons with RADISH-mediated ARM consolidation (Figs 2A and S3C). However, only the output from αβs neurons among three subsets of αβ neurons is required for aversive memory retrieval [35]. Whether the αβs neurons are the only aversive ARM substrate of RADISH remains to be identified.

APL and DPM neurons are two pairs of modulatory neurons broadly innervating the ipsilateral MB, although the DPM neuron’s fiber is lacking in the posterior part of pedunculus and the calyx [15, 20, 36, 37]. Broad, extensive fiber and non-spiking feature [38] allow these two pairs of neurons to have multiple functional roles through different types of neurotransmission [20–22, 36, 37, 39–41]. The APL neuron has been shown to receive odor information from the MB neurons and provide GABAergic feedback inhibition as the *Drosophila* equivalent of a group of the honeybee GABAergic feedback neurons [14, 42]. This feedback inhibition has been proposed to maintain sparse, decorrelated odor coding by suppressing the neuronal activity of MB neurons [14], which can be somewhat linked to the mutual suppression relation with conditioned odor and the facilitation of reversal learning [36, 43]. Interestingly, Pitman et al. proposed that the feedback inhibition from APL neurons sustains the labile appetitive ASM based on *shibire* manipulation [44]. Since *shibire* manipulation can impact small vesicle release, and APL neurons have been demonstrated to co-release at least GABA and octopamine [22, 36], it might worth conducting GABA-specific manipulation in APL neurons to confirm the role in appetitive ASM. For aversive olfactory memory, acute RNAi-mediated knockdown of *Gad1* in APL neurons had no effect on 3-h memory [22]. Instead, the octopamine synthesis enzyme mutant, *Tβh*^*nM18*^, knockdown of *Tβh* in APL neurons, the octopamine receptor mutant, *PBac{WH}octβ2R*^*f05679*^, and knockdown of *octβ2R* in α′β′ neurons all phenocopied the 3-h ARM impairment caused by *shibire*-mediated neurotransmission blockade in APL neurons [22] (see also Fig 2B). Together with the serotonergic DPM–αβ neurons circuit [21], we favor a model that two sets of triple-layered parallel circuits, octopaminergic APL–α′β′–MBON-β′2mp and serotonergic DPM–αβ–MBON-β2β′2a, additively contribute to 3-h aversive ARM.

**Fig 6:**
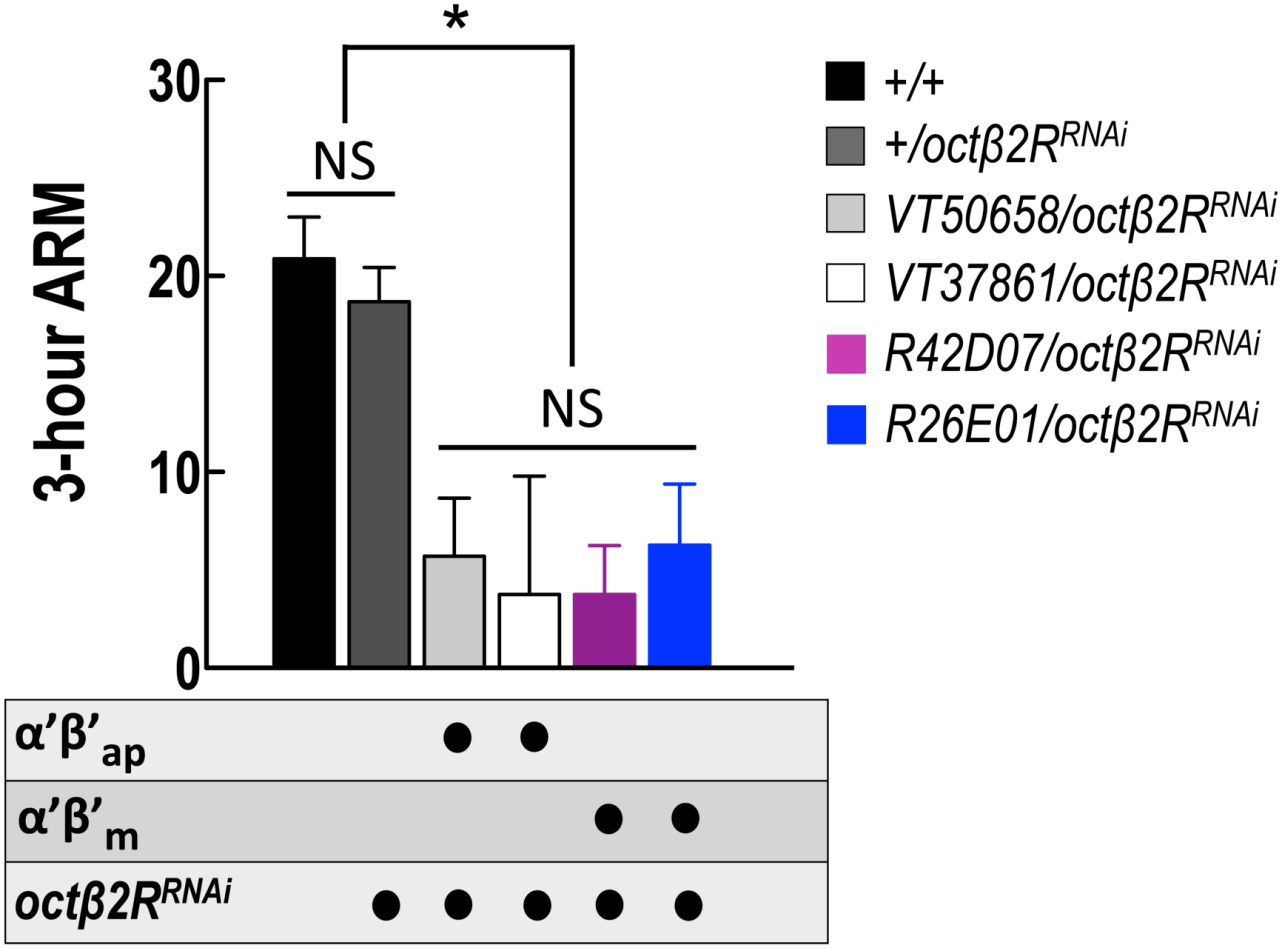
*octβ2R* expression in both MB α′β′ap and α′β′m neurons is required for 3-h ARM consolidation. Knockdown of *octβ2R* in MB α′β′ap or α′β′m neurons impaired ARM. Each value represents mean ±SEM (N = 16, 16, 10, 8, 8, and 8 from left to right bars; *VT50658/octβ2R*^*RNAi*^: *P = 0.0001 as compared to +/+ and +/*octβ2R*^*RNAi*^ controls, *VT37861/octβ2R*^*RNAi*^: *P = 0.0012 as compared to +/+ and +/*octβ2R*^*RNAi*^ controls, *R42D07/octβ2R*^*RNAi*^: *P < 0.0001 as compared to +/+ and +/*octβ2R*^*RNAi*^ controls, and *R26E01/octβ2R*^*RNAi*^: *P = 0.0005 as compared to +/+ and +/*octβ2R*^*RNAi*^ controls, ANOVA followed by Tukey’s test; NS, not significant: *P* = 0.4743 for +/+ and +/ *octβ2R*^*RNAi*^, *t*-test; *P* = 0.9516 for *VT50658/octβ2R*^*RNAi*^, *VT37861/octβ2R*^*RNAi*^, *R42D07/octβ2R*^*RNAi*^, and *R26E01/octβ2R*^*RNAi*^, ANOVA). Genotypes were as follows: (1) *+/+*, (2) *+/+; +/UAS-octβ2R*^*RNAi*^*(v104524)*, (3) *+/+; VT50658-GAL4/UAS-octβ2R*^*RNAi*^*(v104524)*, (4) *+/+; VT37861-GAL4/UAS-octβ2R*^*RNAi*^*(v104524)*, (5) *+/+; R42D07-GAL4/UAS-octβ2R*^*RNAi*^*(v104524)*, (6) *+/+; R26E01-GAL4/UAS-octβ2R*^*RNAi*^*(v104524)*.

Although our data showed that 3-h ARM consolidation requires recurrent output from α′β′ap neurons but not from α′β′m neurons (Fig 5), RNAi-mediated knockdown of *octβ2R* in α′β′ap or α′β′m neurons impaired ARM (Fig 6), suggesting that Octβ2R functions for normal ARM expression in the entire population of α′β′ neurons. On the other hand, neuronal activity during memory consolidation is naturally more quiescent than that during memory retrieval, and the *shibire*-mediated neurotransmission blockade requires an exhaustion of already-docked vesicles. Together with the unfavorable performance for experiments blocking the output from α′β′m neurons during consolidation (left panels of Fig 5C and 5D), we cannot exclude the possibility that output from α′β′m neurons is also required for ARM during consolidation. Alternatively, octopamine signaling may also be involved in ARM retrieval.

## Materials and Methods

### Fly stocks

*Drosophila melanogaster* were raised on standard cornmeal food at 25°C and 70% relative humidity on a 12 h:12 h light:dark cycle. The “Cantonized” w^1118^ w(CS10) was used as the wild-type control. The *UAS-shi*^*ts*^ line used in this study has multiple insertions on the third chromosome. *UAS-Dscam[17*.*1]*::*GFP* flies were obtained from Tzumin Lee. *elav-GAL4;+;tub-GAL80*^*ts*^ flies were obtained from Hsueh-Cheng Chiang. The *UAS-DenMark* flies have been described [45]. The *C305a-GAL4* and *C320-GAL4* have also been described [16, 27]. The *1471-GAL4*, *C739-GAL4*, *R42D07-GAL4*, *R26E01-GAL4*, and *tub-GAL80*^*ts*^ were obtained from the Bloomington Stock Center. *VT49246-GAL4*, *VT37861-GAL4*, *VT50658-GAL4*, *VT41043-GAL4*, and *VT0765-GAL4* were obtained from the Vienna *Drosophila* Resource Center, Vienna Tile (VT). *UAS-radish*^*RNAi*^*(v39931)* and *UAS-VGlut*^*RNAi*^*(v104324)* were obtained from the Vienna *Drosophila* RNAi Center; *VT30604-GAL4*, *VT57244-GAL4*, and *UAS-octβ2R*^*RNAi*^*(v104524)* have been described [22].

### Whole-mount immunostaining

Fly brains were counterstained with the mouse 4F3 anti-discs large (DLG) monoclonal antibody to label all neuronal synapses. The brains were dissected in isotonic PBS and immediately transferred to 4% paraformaldehyde in PBS on ice for a 20-min fixation period. Fixed brain samples were incubated in PBS containing 2% Triton X-100 and 10% normal goat serum (NGS) for 2 h. During the 2-h penetration and blocking period, the brain samples were also subjected to a degassing procedure. Thereafter the brain samples were incubated in a dilution buffer (PBS containing 0.25% Triton X-100, 1% NGS) containing 1:10 mouse anti-DLG monoclonal antibody (Developmental Studies Hybridoma Bank, University of Iowa) at 25°C for one day. After washing in PBS-T three times, the samples were incubated in 1:200 biotinylated goat anti-mouse IgG (Molecular Probes) at 25°C for one day. Next, the brain samples were washed and incubated in 1:500 Alexa Fluor 635 streptavidin or 1:500 Alexa Fluor 488 streptavidin (Molecular Probes) at 25°C overnight. After extensive washing, the brain samples were cleared and mounted in *FocusClear* (CelExplorer) for confocal imaging.

### Confocal microscopy

Sample brains were imaged under a Zeiss LSM 700 confocal microscope with either a 40× C-Apochromat water-immersion objective lens for whole-brain images (N.A. value, 1.2; working distance, 220 μm) or a 63× glycerine-immersion objective lens for horizontal, sagittal, and frontal cross sections (N.A. value, 1.4; working distance, 170 μm). To overcome the limited field of view, some samples were imaged twice, one for each hemisphere, with overlap in between. We then stitched the two parallel image stacks into a single dataset on-line with ZEN software, using the overlapping region to align the two stacks.

### Behavioral assay

Groups of approximately 100 flies were exposed first to one odor (the conditioned stimulus, CS+; 3-octanol or 4-methyl-cyclohexanol) paired with 12 × 1.5-s pulses of 75-V DC electric shock presented at 5-s interpulse intervals. This was followed by the presentation of a second odor (CS–; 4-methyl-cyclohexanol or 3-octanol) without electric shock. In the testing phase, the flies were presented with a choice between CS+ and CS– odors in a T-maze for 2 min. At the end of this 2-min period, the flies were trapped in each T-maze arm were anesthetized and counted. From the distribution of flies between the 2 arms, the performance index (PI) was calculated as the number of flies avoiding the shocked odor (CS+) minus the number avoiding the non-shocked odor (CS–), divided by the total number of flies and multiplied by 100. If the flies did not learn, they were distributed equally between the 2 arms; hence, the calculated PI was 0. However, if all flies avoided the shock-paired odor and were distributed 0:100 between the CS+ and CS– in the T-maze, the PI was 100. To assess learning, performance was measured immediately after training. To evaluate intermediate-term memory, testing was performed 3 h after training. ARM was assayed as 3-h memory, after a 2-min cold shock was presented at 2 h post-training (1 h before testing) by placing a plastic vial containing trained flies in ice water. A brief cold shock, which completely erases short-term memory and labile ASM, leaves only ARM. For the *shi*^*ts*^ experiments, flies were kept at 23°C throughout development. After eclosion, flies were kept at 23°C prior to shifting to 31°C, as indicated by the schematic diagrams above each behavioral graph in the figures. For the adult-stage-specific RNAi-mediated knockdown of *radish* with *tub-GAL80*^*ts*^, flies were kept at 18°C until eclosion and then shifted to 30°C for 7 days before training. The 3-h ARM assay was also performed at 30°C. Control flies were kept at 18°C throughout the experiment.

### Statistical analysis

All raw data were analyzed parametrically with Prism 5.0 software (GraphPad). Because of the nature of their mathematical derivation, performance indices were distributed normally. Hence, the data with more than two groups were evaluated by one-way analysis of variance (ANOVA) and Tukey’s multiple comparisons tests. Data with only two groups were evaluated by paired *t*-test. Definition of statistical significant difference was set as *P* < 0.05. All data were presented as mean ± SEM.

### MB neuron counting

Equal numbers of males and females for each GAL4 line were used for analysis. GAL4 expression patterns were reported by two copies of 5X*UAS-mCD8*::*GFP* reporter, and the MB structure was counterstained with DLG antibody. Each MB neuron labeled by GFP was manually marked with a landmark sphere in the *Amira* software, and the total number of landmark spheres in each hemisphere was calculated.

### Odor-avoidance and shock-avoidance assays

All flies were shifted to the restrictive temperature of 31°C for 15 min before the odor- and shock avoidance tests. For odor avoidance, groups of roughly 100 untrained flies received a 2-min test trial in the T-maze. Different groups were given a choice between either OCT or MCH versus “fresh” room air. The odor avoidance index was calculated as the number of flies in the fresh room air tube minus the number in the odor tube, divided by the total number of flies and multiplied by 100. For shock-avoidance, groups of approximately 100 untrained flies received a 2-min test trial in the T-maze. Each arm of the T-maze contained an electric shock grid, however, and different groups of flies were given a choice between shock and no shock. The shock avoidance index was calculated as the number of flies in the non-shocked grid minus the number in the shocked grid, divided by the total number of flies, and multiplied by 100.

### Quantitative PCR (qPCR)

The effectiveness of the *UAS-radish*^*RNAi*^*(v39931)* and *UAS-VGlut*^*RNAi*^*(v104324)* lines were verified with qPCR. Flies for qPCR were generated by crossing *elav-GAL4* or *elav-GAL4; +; tub-Gal80*^*ts*^ virgin flies with either wild type males or *UAS-VGlut*^*RNAi*^ males or *UAS-radish*^*RNAi*^ males. RNA in the isolated heads of adult flies or in whole 3^rd^ instar larvae was extracted with TRIZOL Reagent (Invitrogen, Life Technologies, USA). The extracted RNA was used to synthesize first-strand cDNA with High-Capacity cDNA Reverse Transcription Kits (Applied Biosystems, USA). RNA expression levels were quantified by qPCR (StepOnePlus^™^ System, Applied Biosystems). qPCR and quantitative measurements were performed with the SYBR-Green PCR-Master Mix (Applied Biosystems).

## Supporting Information

S1 Fig Neurotransmissions from MB γ neurons are required for 3-h anesthesia-sensitive memory (ASM) retrieval. Neurotransmission was blocked by keeping *shi*^*ts*^ flies at restrictive temperature (31°C) starting 15 min prior to and during testing. Each value represents mean ±SEM (left panel: *P < 0.0001, N = 7 for each bar, ANOVA followed by Tukey’s test; right panel: *P* = 0.3179, N = 8 for each bar, ANOVA).
(TIF)

S2 Fig Behavioral control experiments in *MB-GAL4s* combined with *UAS-shi*^*ts*^. (A1–A5) 3-h memory at permissive temperature (23°C) in *VT44966-GAL4 > UAS-shi*^*ts*^, *VT49246-GAL4 > UAS-shi*^*ts*^, *VT30604-GAL4 > UAS-shi*^*ts*^, *VT57244-GAL4 > UAS-shi*^*ts*^, and *C739-GAL4;VT30604-GAL4 > UAS-shi*^*ts*^ flies. Each value represents mean ± SEM, N = 8 for each bar (A1: *P* = 0.7355, ANOVA; A2: *P* = 0.2413, ANOVA; A3: *P* = 0.9157, ANOVA; A4: *P* = 0.9653, ANOVA; A5: *P* = 0.7587, ANOVA). (B1–B5) Olfactory acuity to 3-octanol (OCT) or 4-methylcyclohexanol (MCH) at restrictive temperature (31°C) in *VT44966-GAL4 > UAS-shi*^*ts*^, *VT49246-GAL4 > UAS-shi*^*ts*^, *VT30604-GAL4 > UAS-shi*^*ts*^, *VT57244-GAL4 > UAS-shi*^*ts*^, and *C739-GAL4;VT30604-GAL4 > UAS-shi*^*ts*^ flies. Each value represents mean ± SEM, N = 6 for each bar (B1: *P* = 0.6771 for OCT and *P* = 0.7778 for MCH, ANOVA; B2: *P* = 0.8648 for OCT and *P* = 0.9401 for MCH, ANOVA; B3: *P* = 0.8942 for OCT and *P* = 0.9609 for MCH, AVOVA; B4: *P* = 0.7691 for OCT and *P* = 0.7306 for MCH, ANOVA; B5: *P* = 0.9846 for OCT and *P* = 0.9098 for MCH, ANOVA). (C) Electrical shock avoidance at restrictive temperature (31°C) in *VT44966-GAL4 > UAS-shi*^*ts*^, *VT49246-GAL4 > UAS-shi*^*ts*^, *VT30604-GAL4 > UAS-shi*^*ts*^, *VT57244-GAL4 > UAS-shi*^*ts*^, and *C739-GAL4;VT30604-GAL4 > UAS-shi*^*ts*^ flies. Each value represents mean ± SEM (*P* = 0.7441, N = 6 for each bar, ANOVA).
(TIF)

S3 Fig Effectiveness of *UAS-radish*^*RNAi*^ and behavioral control experiments of *rsh*^*1*^*;+;VT49246-GAL4 > UAS-shi*^*ts*^ and *rsh*^*1*^*;+;VT30604-GAL4 > UAS-shi*^*ts*^ flies. (A) Effectiveness of *UAS-radish*^*RNAi*^ line used in this study. Quantitative PCR shows that the amount of *radish* mRNA in the *elav-GAL4 > UAS-radish*^*RNAi*^*(v39931)* (*elav/rsh*^*RNAi*^) flies was less than that in the control *elav-GAL4/+* (*elav/+*) flies. The results were normalized to the relative amount of 60S ribosomal protein L32 (RpL32). Each value represents mean ± SEM. (N = 3). Forward and reverse primers used were 5′-AGTTCCACAACGCTGATATTCC-3′ and 5′- GGGGTGGGCATAGTGATCTT-3′, respectively. (B) Adult-stage-specific knockdown of *radish*. Flies were incubated at 18°C until eclosion and then shifted to 30°C for 7 day. Quantitative PCR shows that the amount of *radish* mRNA in the *elav-GAL4; +; tub-GAL80*^*ts*^ *> UAS-radish*^*RNAi*^*(v39931)* (*elav;+;tub-GAL80*^*ts*^*/rsh*^*RNAi*^) flies was not changed at the 3^rd^ instar larvae stage but was reduced at adult stage as compared to the control *elav-GAL4; +; tub-GAL80*^*ts*^*/+* (*elav;+;tub-GAL80*^*ts*^*/+*) groups. The results were normalized to the relative amount of 60S ribosomal protein L32 (RpL32). Each value represents mean ± SEM. (N = 13 for larvae and N = 4 for adults). Forward and reverse primers used were 5′-AGTTCCACAACGCTGATATTCC-3′ and 5′- GGGGTGGGCATAGTGATCTT-3′, respectively. (C) Adult-stage-specific knockdown of *radish* in MB γ neurons did not affect ARM. Flies were incubated at 18°C until eclosion and then shifted to 30°C for 7 day and performed the experiments at 30°C. Each value represents mean ± SEM (*P* = 0.4352, N = 8 for each bar, ANOVA). Genotypes were as follows: (1) *tub-GAL80*^*ts*^*/+; VT44966-GAL4/+*, (2) *+/UAS-radish*^*RNAi*^*(v39931); +/+*, (3) *tub-GAL80*^*ts*^*/UAS-radish*^*RNAi*^*(v39931); VT44966-GAL4/+*. (D) Olfactory acuity to OCT or MCH and electrical shock avoidance at restrictive temperature (31°C) in (1) *rsh*^*1*^*; +/+; +/UAS-shi*^*ts*^, (2) *rsh*^*1*^*; +/+; VT49246-GAL4/+*, (3) *rsh*^*1*^*; +/+; VT30604-GAL4/+*, (4) *rsh*^*1*^*; +/+; VT49246-GAL4/UAS-shi*^*ts*^, and (5) *rsh*^*1*^*; +/+; VT30604-GAL4/UAS-shi*^*ts*^ flies. Each value represents mean ± SEM (*P* = 0.6492 for OCT, *P* = 0.6765 for MCH and *P* = 0.8690 for shock response, N = 6 for each bar, ANOVA).
(TIF)

S4 Fig Dendritic labeling with DenMark in MBON-β2β′2a or MBON-β′2mp neurons and behavioral control experiments in blocking neurotransmissions from MBON-β2β′2a or MBON-β′2mp neurons. (A) Sub-regional dendritic distributions of MBON-β2β′2a and MBON-β′2mp neurons with *DenMark* positive signals (red). Brains were counterstained with DLG antibody (green). The scale bar represents 10 μm. Genotypes: (1) *+/UAS-DenMark; VT0765-GAL4/+*, (2) *+/UAS-DenMark; VT41043-GAL4/+*. (B) Olfactory acuity to OCT or MCH and electrical shock avoidance at restrictive temperature (31°C) in *VT0765-GAL4 > UAS-shi*^*ts*^ and *VT41043-GAL4 > UAS-shi*^*ts*^ flies. Each value represents mean ± SEM (*P* = 0.8165 for OCT, *P* = 0.5913 for MCH, and *P* = 0.9068 for shock response, N = 6 for each bar, ANOVA).
(TIF)

S5 Fig Adult-stage-specific knockdown of *VGlut* in MBON-β2β′2a or MBON-β′2mp neurons disrupted ARM. (A) Quantitative PCR shows that the amount of target mRNA in the *elav-GAL4 >UAS-VGlut*^*RNAi*^*(v104324)* (*elav/VGlut*^*RNAi*^) flies was less than that in the control *elav-GAL4/+* (*elav/+*) flies. The results were normalized to the relative amount of 60S ribosomal protein L32 (RpL32). Each value represents mean ± SEM. (N = 3). Forward and reverse primers used were 5′-CCTTCGGCATGAGGTGCAATA-3′ and 5′-CGAGTCCACATGGCTCTCC-3′, respectively. (B) Inducible RNAi-mediated knockdown of *VGlut* expression in MBON-β2β′2a or MBON-β′2mp neurons in the adult stage disrupted ARM. Each value represents mean ± SEM. (left panel: *P* = 0.9391, N = 8 for each bar, ANOVA; right panel: *P = 0.0028 for *VT0765/VGlut*^*RNAi*^*; tub-GAL80*^*ts*^ and *P = 0.0040 for *VT41043/VGlut*^*RNAi*^*; tub-GAL80*^*ts*^ as compared to the control flies, N = 12 for each bar, ANOVA followed by Tukey’s test). Genotypes: (1) *+/+*, (2) *+/UAS-VGlut*^*RNAi*^*(v104324); +/tub-GAL80*^*ts*^, (3) *+/+; VT0765-GAL4/+*, (4) *+/+; VT41043-GAL4/+*, (5) *+/UAS-VGlut*^*RNAi*^*(v104324); VT0765-GAL4/tub-GAL80*^*ts*^, (6) *+/UAS-VGlut*^*RNAi*^*(v104324); VT41043-GAL4/tub-GAL80*^*ts*^.
(TIF)

S6 Fig Blocking neurotransmissions from *C305a-GAL4* or *C320-GAL4* neurons during 3-h memory consolidation or retrieval. (A1–A4) The MB structure (A1), and single horizontal, sagittal, and frontal confocal cross sections of the MB lobes selected for analyses at the level of yellow lines or red square region (A2–A4). (B1–B4) *C305a-GAL4* expresses in MB α′β′ap and α′β′m neurons (green). The brain was immunostained with DLG antibody (magenta). Genotype was as follows: *C305a-GAL4/UAS-mCD8*::*GFP; +/UAS-mCD8*::*GFP*. (C1–C4) *C320-GAL4* expresses in MB α′β′ap neurons (green). The brain was immunostained with DLG antibody (magenta). Genotype was as follows: *C320a-GAL4/UAS-mCD8*::*GFP; +/UAS-mCD8*::*GFP*. Neurotransmission was blocked by keeping *shi*^*ts*^ flies at restrictive temperature (31°C) for 1 h immediately after training (D1 and E1) or starting 15 min prior to and during testing (D2 and E2). Each value represents mean ± SEM (D1: *P = 0.0044, N = 8 for each bar, ANOVA followed by Tukey’s test; D2: *P* = 0.7603, N = 8 for each bar, ANOVA; E1: *P = 0.0001, N = 6 for each bar, ANOVA followed by Tukey’s test; E2: *P* = 0.0575, N = 8 for each bar, ANOVA). Genotypes for D1 and D2: (1) *+/+*, (2) *C305a-GAL4/+; +/+*, (3) *+/+; +/UAS-shi*^*ts*^, (4) *C305a-GAL4/+; +/UAS-shi*^*ts*^. Genotypes for E1 and E2: (1) *+/+*, (2) *C320-GAL4/+; +/+*, (3) *+/+; +/UAS-shi*^*ts*^, (4) *C320-GAL4/+; +/UAS-shi*^*ts*^.
(TIF)

S7 Fig Behavioral control experiments of MB α′β′ap- and α′β′m- *GAL4s* combined with *UAS-shi*^*ts*^. (A1–A4) Olfactory acuity to OCT or MCH at restrictive temperature (31°C) in *VT50658-GAL4 > UAS-shi*^*ts*^, *VT37861-GAL4 > UAS-shi*^*ts*^, *R42D07-GAL4 > UAS-shi*^*ts*^, and *R26E01-GAL4 > UAS-shi*^*ts*^ flies. Each value represents mean ± SEM, N = 6 for each bar (A1: *P* = 0.7098 for OCT and *P* = 0.9220 for MCH, ANOVA; A2: *P* = 0.9418 for OCT and *P* = 0.2658 for MCH, ANOVA; A3: *P* = 0.9555 for OCT and *P* = 0.6939 for MCH, ANOVA; A4: *P* = 0.9372 for OCT and *P* = 0.7184 for MCH, ANOVA). (B) Electrical shock avoidance at restrictive temperature (31°C) in *VT50658-GAL4 > UAS-shi*^*ts*^, *VT37861-GAL4 > UAS-shi*^*ts*^, *R42D07-GAL4 > UAS-shi*^*ts*^, and *R26E01-GAL4 > UAS-shi*^*ts*^ flies. Each value represents mean ± SEM (*P* = 0.6855, N = 6 for each bar, ANOVA).
(TIF)
