# Supplementary figures and images for: Additive Expression of Consolidated Memory through Drosophila Mushroom Body Subsets
Source: PLoS Genet. 2016 May 19;12(5):e1006061. doi: 10.1371/journal.pgen.1006061 (PMC4873240; doi:10.1371/journal.pgen.1006061)

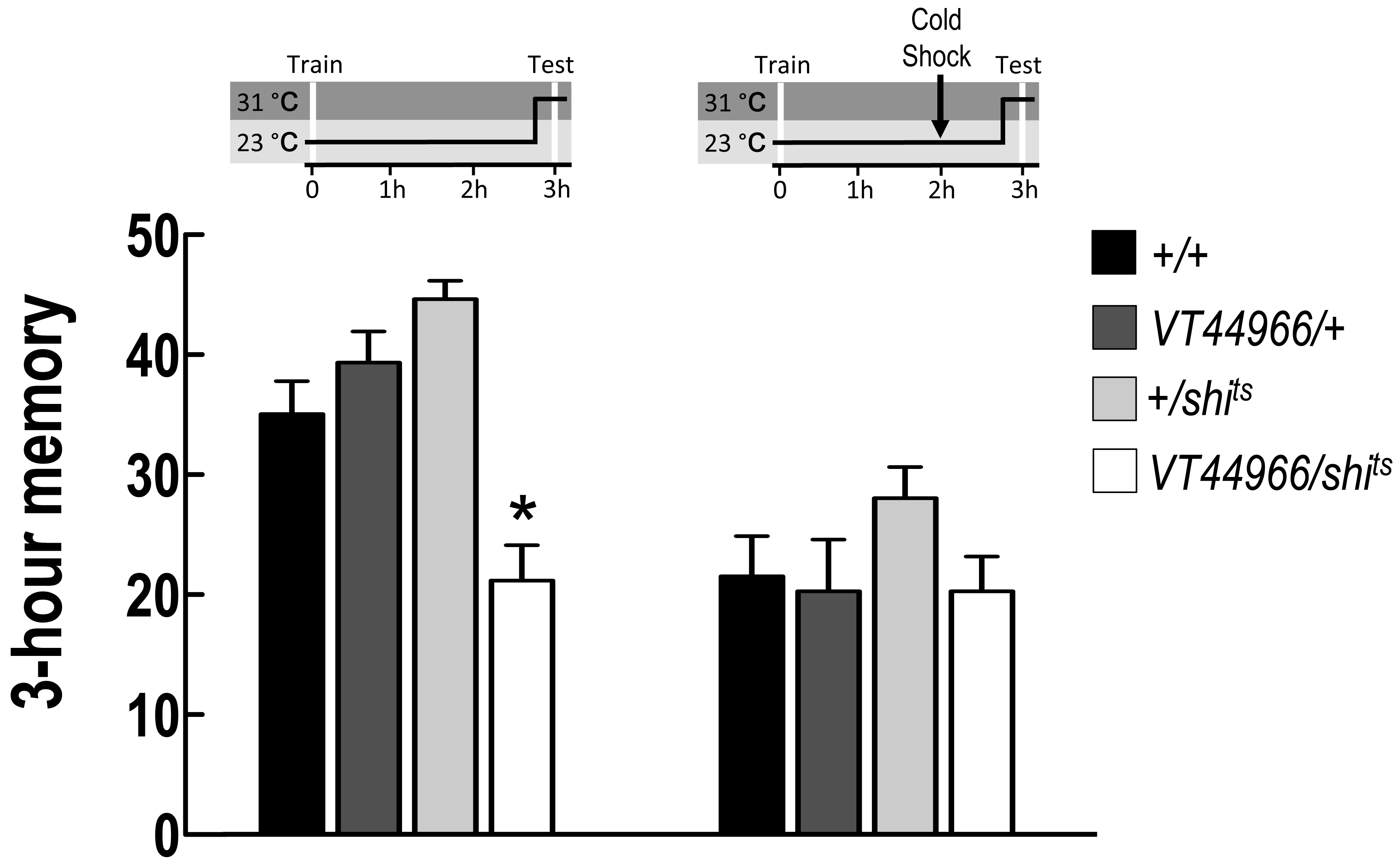

Supplement: S1 Fig — Neurotransmission was blocked by keeping shits flies at restrictive temperature (31°C) starting 15 min prior to and during testing. Each value represents mean ±SEM (left panel: *P < 0.0001, N = 7 for each bar, ANOVA followed by Tukey’s test; right panel: P = 0.3179, N = 8 for each bar, ANOVA). (TIF) [file pgen.1006061.s001.tif]

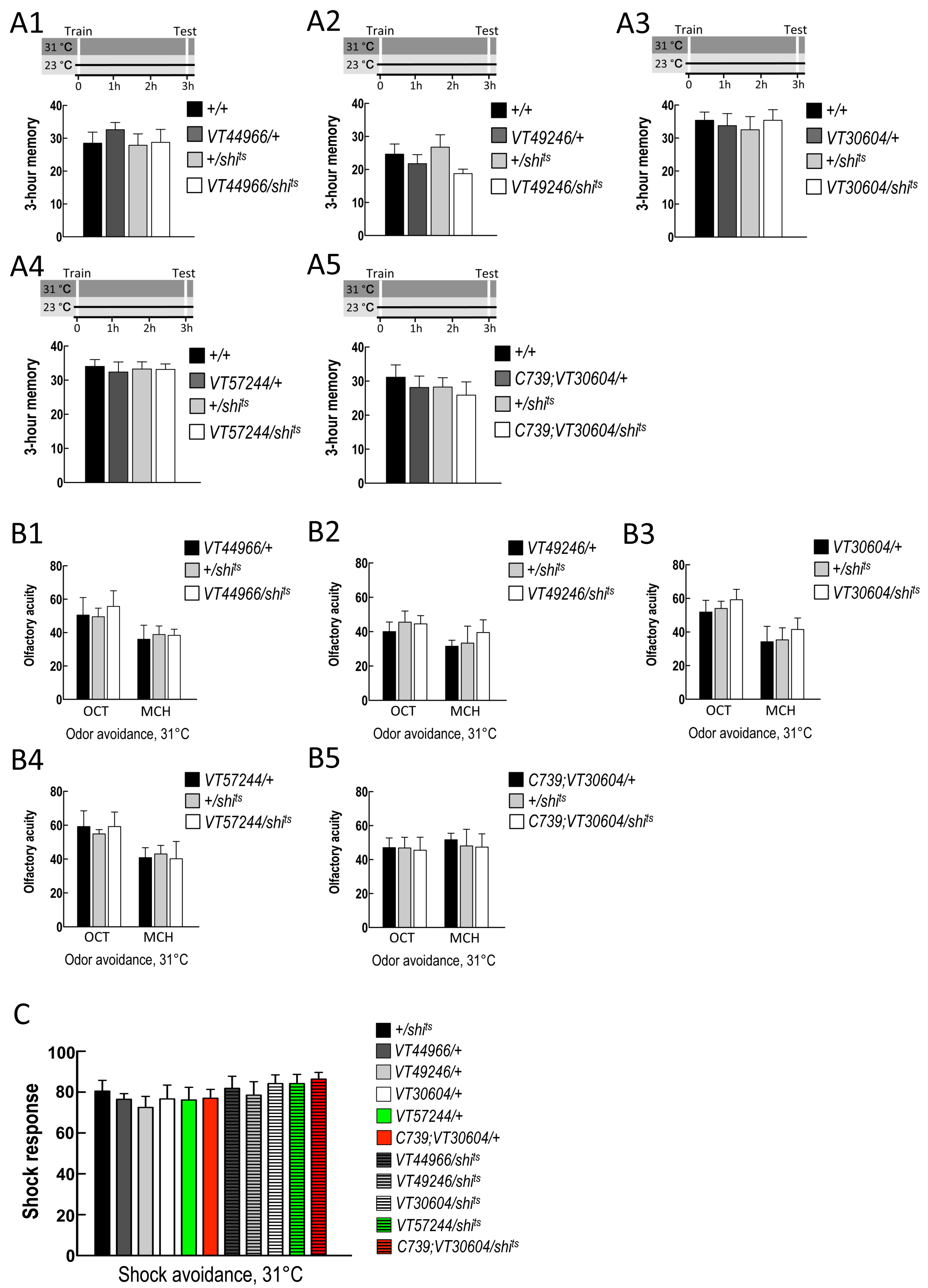

Supplement: S2 Fig — (A1–A5) 3-h memory at permissive temperature (23°C) in VT44966-GAL4 > UAS-shits, VT49246-GAL4 > UAS-shits, VT30604-GAL4 > UAS-shits, VT57244-GAL4 > UAS-shits, and C739-GAL4;VT30604-GAL4 > UAS-shits flies. Each value represents mean ± SEM, N = 8 for each bar (A1: P = 0.7355, ANOVA; A2: P = 0.2413, ANOVA; A3: P = 0.9157, ANOVA; A4: P = 0.9653, ANOVA; A5: P = 0.7587, ANOVA). (B1–B5) Olfactory acuity to 3-octanol (OCT) or 4-methylcyclohexanol (MCH) at restrictive temperature (31°C) in VT44966-GAL4 > UAS-shits, VT49246-GAL4 > UAS-shits, VT30604-GAL4 > UAS-shits, VT57244-GAL4 > UAS-shits, and C739-GAL4;VT30604-GAL4 > UAS-shits flies. Each value represents mean ± SEM, N = 6 for each bar (B1: P = 0.6771 for OCT and P = 0.7778 for MCH, ANOVA; B2: P = 0.8648 for OCT and P = 0.9401 for MCH, ANOVA; B3: P = 0.8942 for OCT and P = 0.9609 for MCH, AVOVA; B4: P = 0.7691 for OCT and P = 0.7306 for MCH, ANOVA; B5: P = 0.9846 for OCT and P = 0.9098 for MCH, ANOVA). (C) Electrical shock avoidance at restrictive temperature (31°C) in VT44966-GAL4 > UAS-shits, VT49246-GAL4 > UAS-shits, VT30604-GAL4 > UAS-shits, VT57244-GAL4 > UAS-shits, and C739-GAL4;VT30604-GAL4 > UAS-shits flies. Each value represents mean ± SEM (P = 0.7441, N = 6 for each bar, ANOVA). (TIF) [file pgen.1006061.s002.tif]

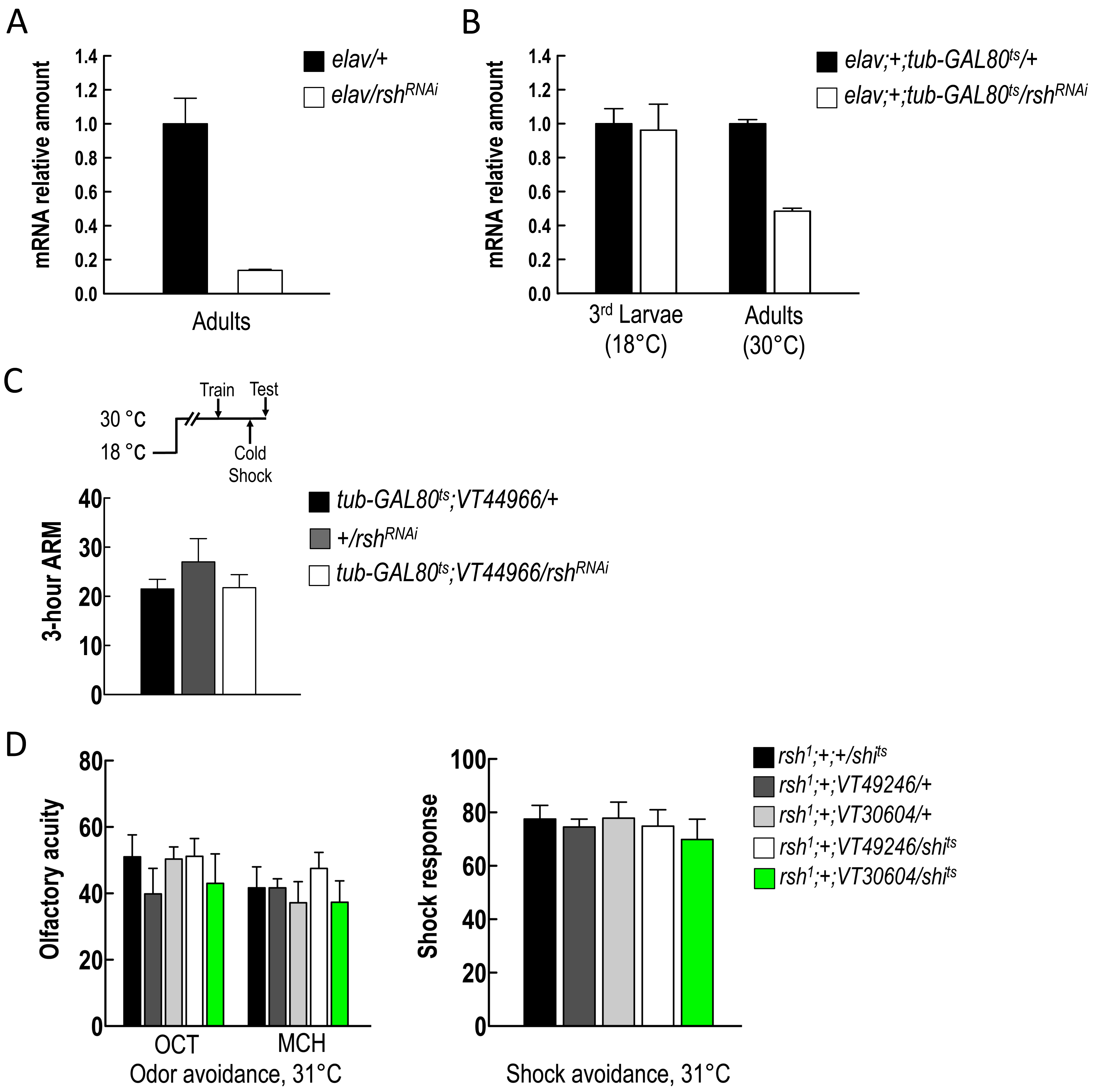

Supplement: S3 Fig — (A) Effectiveness of UAS-radishRNAi line used in this study. Quantitative PCR shows that the amount of radish mRNA in the elav-GAL4 > UAS-radishRNAi(v39931) (elav/rshRNAi) flies was less than that in the control elav-GAL4/+ (elav/+) flies. The results were normalized to the relative amount of 60S ribosomal protein L32 (RpL32). Each value represents mean ± SEM. (N = 3). Forward and reverse primers used were 5′-AGTTCCACAACGCTGATATTCC-3′ and 5′- GGGGTGGGCATAGTGATCTT-3′, respectively. (B) Adult-stage-specific knockdown of radish. Flies were incubated at 18°C until eclosion and then shifted to 30°C for 7 day. Quantitative PCR shows that the amount of radish mRNA in the elav-GAL4; +; tub-GAL80ts > UAS-radishRNAi(v39931) (elav;+;tub-GAL80ts/rshRNAi) flies was not changed at the 3rd instar larvae stage but was reduced at adult stage as compared to the control elav-GAL4; +; tub-GAL80ts/+ (elav;+;tub-GAL80ts/+) groups. The results were normalized to the relative amount of 60S ribosomal protein L32 (RpL32). Each value represents mean ± SEM. (N = 13 for larvae and N = 4 for adults). Forward and reverse primers used were 5′-AGTTCCACAACGCTGATATTCC-3′ and 5′- GGGGTGGGCATAGTGATCTT-3′, respectively. (C) Adult-stage-specific knockdown of radish in MB γ neurons did not affect ARM. Flies were incubated at 18°C until eclosion and then shifted to 30°C for 7 day and performed the experiments at 30°C. Each value represents mean ± SEM (P = 0.4352, N = 8 for each bar, ANOVA). Genotypes were as follows: (1) tub-GAL80ts/+; VT44966-GAL4/+, (2) +/UAS-radishRNAi(v39931); +/+, (3) tub-GAL80ts/UAS-radishRNAi(v39931); VT44966-GAL4/+. (D) Olfactory acuity to OCT or MCH and electrical shock avoidance at restrictive temperature (31°C) in (1) rsh1; +/+; +/UAS-shits, (2) rsh1; +/+; VT49246-GAL4/+, (3) rsh1; +/+; VT30604-GAL4/+, (4) rsh1; +/+; VT49246-GAL4/UAS-shits, and (5) rsh1; +/+; VT30604-GAL4/UAS-shits flies. Each value represents mean ± SEM (P = 0.6492 for OCT, P = 0.6765 for MCH and P = 0.8690 for [file pgen.1006061.s003.tif]

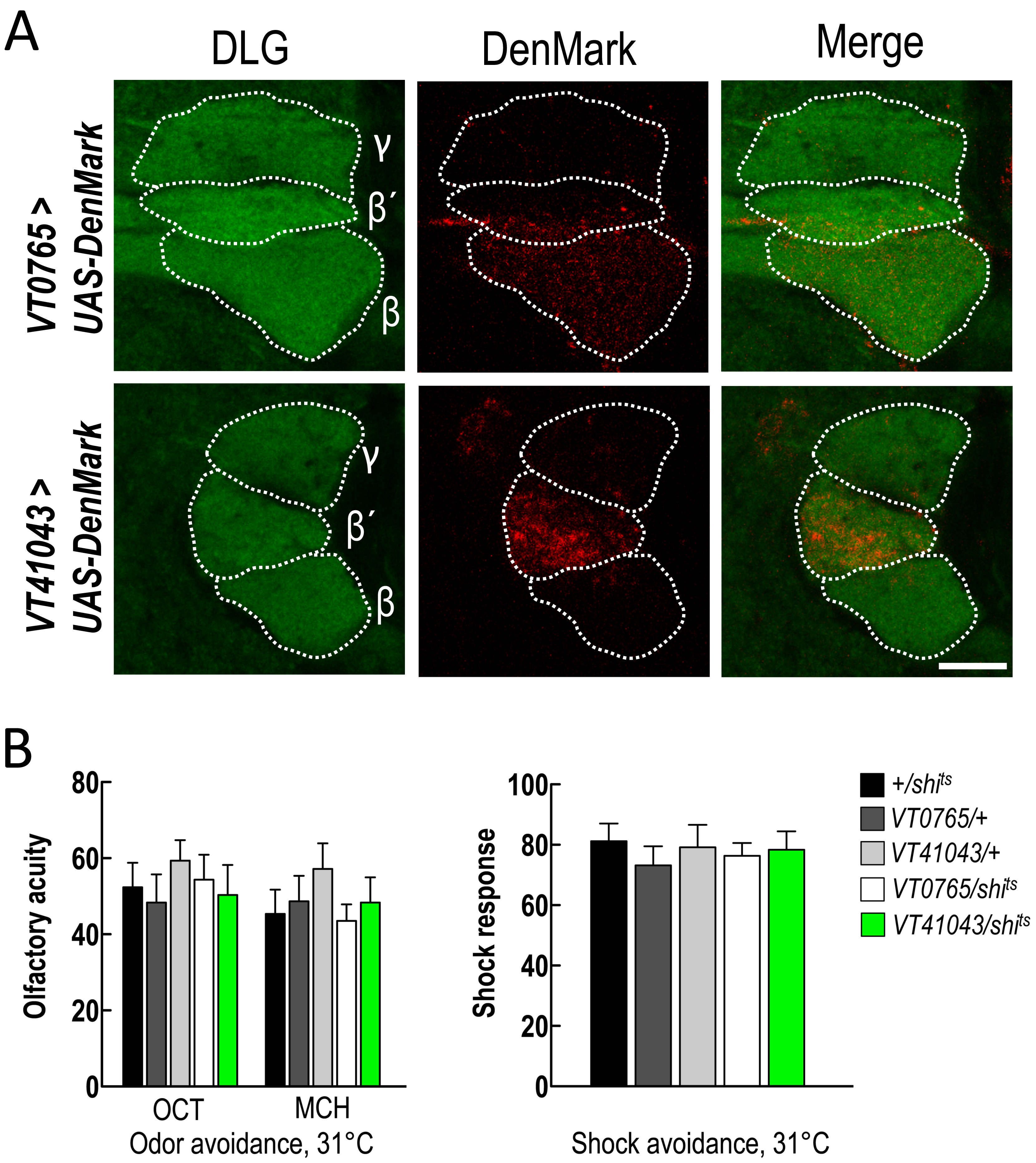

Supplement: S4 Fig — (A) Sub-regional dendritic distributions of MBON-β2β′2a and MBON-β′2mp neurons with DenMark positive signals (red). Brains were counterstained with DLG antibody (green). The scale bar represents 10 μm. Genotypes: (1) +/UAS-DenMark; VT0765-GAL4/+, (2) +/UAS-DenMark; VT41043-GAL4/+. (B) Olfactory acuity to OCT or MCH and electrical shock avoidance at restrictive temperature (31°C) in VT0765-GAL4 > UAS-shits and VT41043-GAL4 > UAS-shits flies. Each value represents mean ± SEM (P = 0.8165 for OCT, P = 0.5913 for MCH, and P = 0.9068 for shock response, N = 6 for each bar, ANOVA). (TIF) [file pgen.1006061.s004.tif]

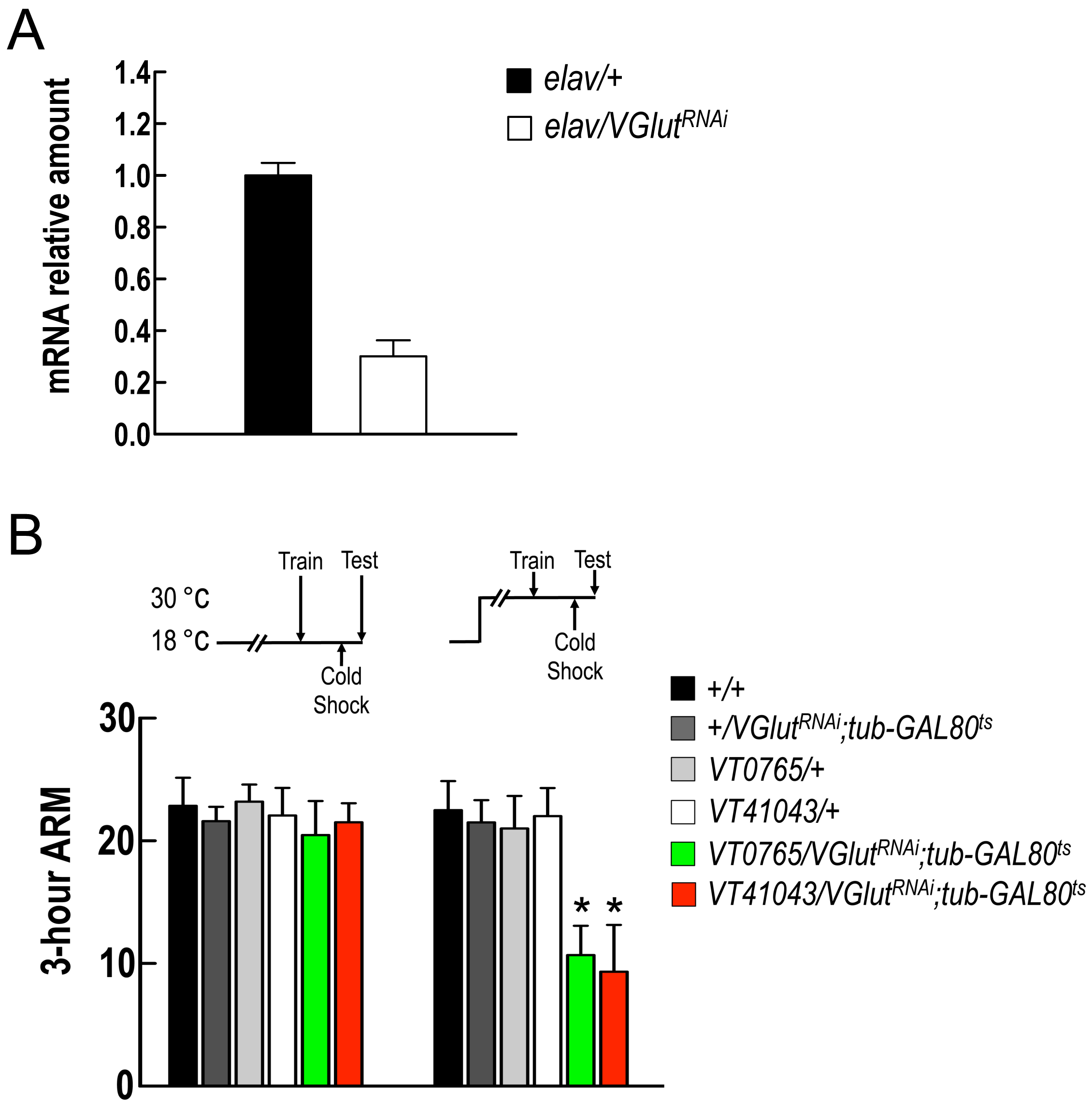

Supplement: S5 Fig — (A) Quantitative PCR shows that the amount of target mRNA in the elav-GAL4 >UAS-VGlutRNAi(v104324) (elav/VGlutRNAi) flies was less than that in the control elav-GAL4/+ (elav/+) flies. The results were normalized to the relative amount of 60S ribosomal protein L32 (RpL32). Each value represents mean ± SEM. (N = 3). Forward and reverse primers used were 5′-CCTTCGGCATGAGGTGCAATA-3′ and 5′-CGAGTCCACATGGCTCTCC-3′, respectively. (B) Inducible RNAi-mediated knockdown of VGlut expression in MBON-β2β′2a or MBON-β′2mp neurons in the adult stage disrupted ARM. Each value represents mean ± SEM. (left panel: P = 0.9391, N = 8 for each bar, ANOVA; right panel: *P = 0.0028 for VT0765/VGlutRNAi; tub-GAL80ts and *P = 0.0040 for VT41043/VGlutRNAi; tub-GAL80ts as compared to the control flies, N = 12 for each bar, ANOVA followed by Tukey’s test). Genotypes: (1) +/+, (2) +/UAS-VGlutRNAi(v104324); +/tub-GAL80ts, (3) +/+; VT0765-GAL4/+, (4) +/+; VT41043-GAL4/+, (5) +/UAS-VGlutRNAi(v104324); VT0765-GAL4/tub-GAL80ts, (6) +/UAS-VGlutRNAi(v104324); VT41043-GAL4/tub-GAL80ts. (TIF) [file pgen.1006061.s005.tif]

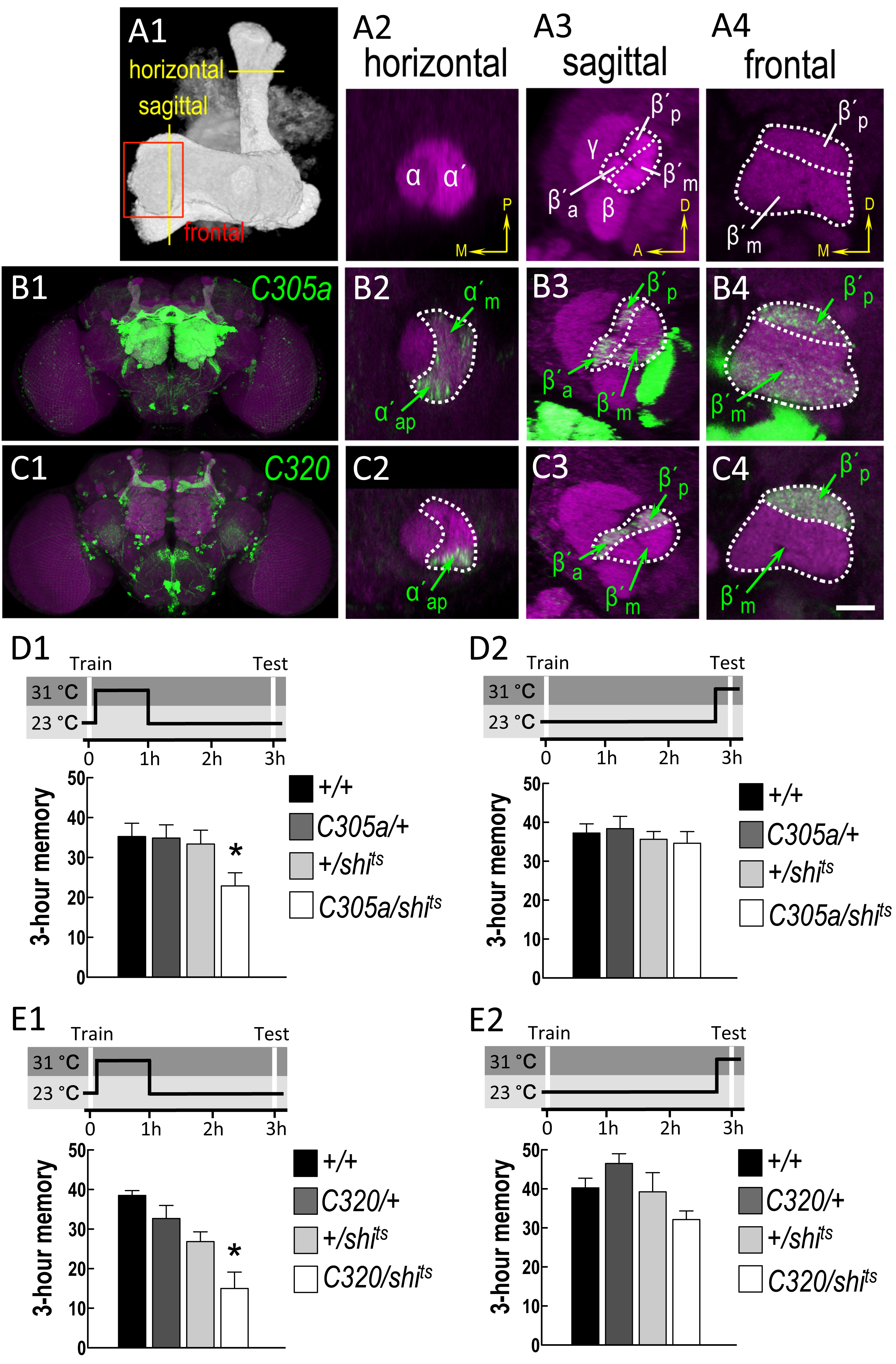

Supplement: S6 Fig — (A1–A4) The MB structure (A1), and single horizontal, sagittal, and frontal confocal cross sections of the MB lobes selected for analyses at the level of yellow lines or red square region (A2–A4). (B1–B4) C305a-GAL4 expresses in MB α′β′ap and α′β′m neurons (green). The brain was immunostained with DLG antibody (magenta). Genotype was as follows: C305a-GAL4/UAS-mCD8::GFP; +/UAS-mCD8::GFP. (C1–C4) C320-GAL4 expresses in MB α′β′ap neurons (green). The brain was immunostained with DLG antibody (magenta). Genotype was as follows: C320a-GAL4/UAS-mCD8::GFP; +/UAS-mCD8::GFP. Neurotransmission was blocked by keeping shits flies at restrictive temperature (31°C) for 1 h immediately after training (D1 and E1) or starting 15 min prior to and during testing (D2 and E2). Each value represents mean ± SEM (D1: *P = 0.0044, N = 8 for each bar, ANOVA followed by Tukey’s test; D2: P = 0.7603, N = 8 for each bar, ANOVA; E1: *P = 0.0001, N = 6 for each bar, ANOVA followed by Tukey’s test; E2: P = 0.0575, N = 8 for each bar, ANOVA). Genotypes for D1 and D2: (1) +/+, (2) C305a-GAL4/+; +/+, (3) +/+; +/UAS-shits, (4) C305a-GAL4/+; +/UAS-shits. Genotypes for E1 and E2: (1) +/+, (2) C320-GAL4/+; +/+, (3) +/+; +/UAS-shits, (4) C320-GAL4/+; +/UAS-shits. (TIF) [file pgen.1006061.s006.tif]

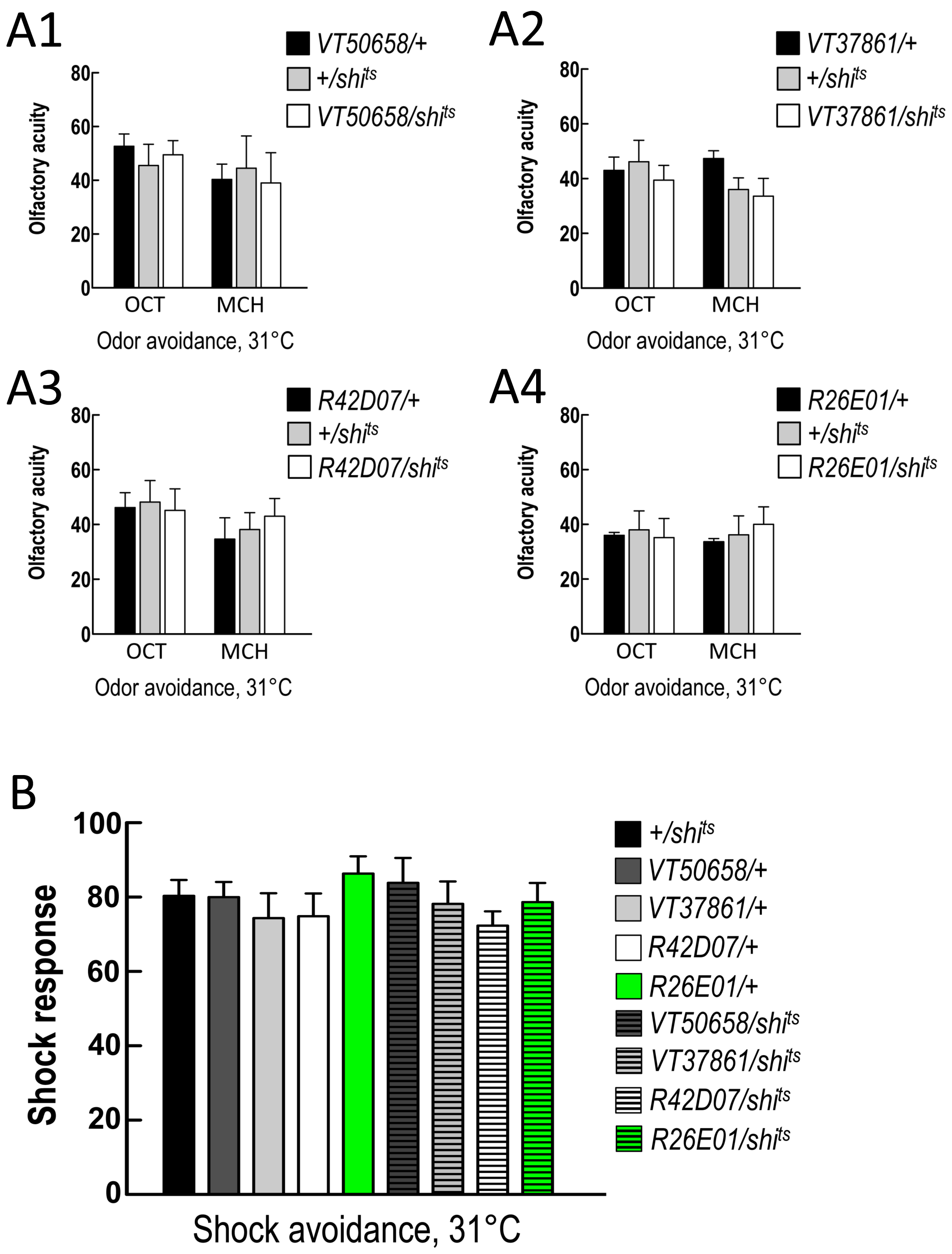

Supplement: S7 Fig — (A1–A4) Olfactory acuity to OCT or MCH at restrictive temperature (31°C) in VT50658-GAL4 > UAS-shits, VT37861-GAL4 > UAS-shits, R42D07-GAL4 > UAS-shits, and R26E01-GAL4 > UAS-shits flies. Each value represents mean ± SEM, N = 6 for each bar (A1: P = 0.7098 for OCT and P = 0.9220 for MCH, ANOVA; A2: P = 0.9418 for OCT and P = 0.2658 for MCH, ANOVA; A3: P = 0.9555 for OCT and P = 0.6939 for MCH, ANOVA; A4: P = 0.9372 for OCT and P = 0.7184 for MCH, ANOVA). (B) Electrical shock avoidance at restrictive temperature (31°C) in VT50658-GAL4 > UAS-shits, VT37861-GAL4 > UAS-shits, R42D07-GAL4 > UAS-shits, and R26E01-GAL4 > UAS-shits flies. Each value represents mean ± SEM (P = 0.6855, N = 6 for each bar, ANOVA). (TIF) [file pgen.1006061.s007.tif]
